# Identification of SRY‐box 30 as an age‐related essential gatekeeper for male germ‐cell meiosis and differentiation

**DOI:** 10.1111/acel.13343

**Published:** 2021-03-15

**Authors:** Fei Han, Li Yin, Xiao Jiang, Xi Zhang, Ning Zhang, Jun‐tang Yang, Wei‐ming Ouyang, Xiang‐lin Hao, Wen‐bin Liu, Yong‐sheng Huang, Hong‐qiang Chen, Fei Gao, Zhong‐tai Li, Qiao‐nan Guo, Jia Cao, Jin‐yi Liu

**Affiliations:** ^1^ Institute of Toxicology College of Preventive Medicine Army Medical University Chongqing China; ^2^ College of Pharmacy and Bioengineering Chongqing University of Technology Chongqing China; ^3^ College of Life Science Henan Normal University Henan China; ^4^ Office of Biotechnology Products Center for Drug Evaluation and Research U.S. Food and Drug Administration Pittsburgh PA USA; ^5^ Department of Pathology Xinqiao Hospital Army Medical University Chongqing China; ^6^ Department of Veterinary and Animal Sciences Faculty of Health and Medical Sciences University of Copenhagen Frederiksberg DK Denmark; ^7^ Department of Urology Daping Hospital Army Medical University Chongqing China

**Keywords:** induced recovery, meiosis arrest, postnatal testis, retinoic acid signalling, SRY‐box 30, zygotene spermatocyte

## Abstract

Although important factors governing the meiosis have been reported in the embryonic ovary, meiosis in postnatal testis remains poorly understood. Herein, we first report that SRY‐box 30 (Sox30) is an age‐related and essential regulator of meiosis in the postnatal testis. Sox30‐null mice exhibited uniquely impaired testis, presenting the abnormal arrest of germ‐cell differentiation and irregular Leydig cell proliferation. In aged Sox30‐null mice, the observed testicular impairments were more severe. Furthermore, the germ‐cell arrest occurred at the stage of meiotic zygotene spermatocytes, which is strongly associated with critical regulators of meiosis (such as *Cyp26b1*, *Stra8* and *Rec8*) and sex differentiation (such as *Rspo1*, *Foxl2*, *Sox9*, *Wnt4* and *Ctnnb1*). Mechanistically, Sox30 can activate *Stra8* and *Rec8*, and inhibit *Cyp26b1* and *Ctnnb1* by direct binding to their promoters. A different Sox30 domain required for regulating the activity of these gene promoters, providing a “fail‐safe” mechanism for Sox30 to facilitate germ‐cell differentiation. Indeed, retinoic acid levels were reduced owing to increased degradation following the elevation of Cyp26b1 in Sox30‐null testes. Re‐expression of Sox30 in Sox30‐null mice successfully restored germ‐cell meiosis, differentiation and Leydig cell proliferation. Moreover, the restoration of actual fertility appeared to improve over time. Consistently, *Rec8* and *Stra8* were reactivated, and *Cyp26b1* and *Ctnnb1* were reinhibited in the restored testes. In summary, *Sox30* is necessary, sufficient and age‐associated for germ‐cell meiosis and differentiation in testes by direct regulating critical regulators. This study advances our understanding of the regulation of germ‐cell meiosis and differentiation in the postnatal testis.

## Graphical abstract

We reported that Sox30‐null mice exhibit uniquely impaired testis with irregular Leydig cell proliferation and abnormal germ‐cell arrest at zygotene, and the observed testicular impairments were more severe as mice aged. Sox30 controls male germ‐cell meiosis by a “fail‐safe” regulation of retinoic acid signalling, and re‐expression of Sox30 in Sox30‐null mice can successfully restore germ‐cell meiosis and differentiation. Moreover, the restoration of actual fertility appeared to improve over time.

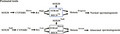

## 1 INTRODUCTION

Meiosis is a programme of two cell divisions (meiosis I and II), with only one round of DNA replication generating haploid gametes. Meiosis I is composed of four phases: prophase I, metaphase I, anaphase I and telophase I. Among these phases, prophase I is the longest phase of meiosis and can be divided into five stages: leptotene, zygotene, pachytene, diplotene and diakinesis. The timing and regulation of mammalian meiosis differ dramatically between sexes (Baltus et al., 2006; Feng et al., 2014; Handel & Schimenti, 2010; Spiller & Bowles, 2015). In mouse embryonic ovary, germ cells switch from mitosis to meiosis around 13.5 days post‐conception (dpc). In mouse testis, meiosis is initiated around 8‐day post‐partum (dpp). Foetal ovarian germ cells respond to soma‐produced retinoic acid (RA), an active metabolite of Vitamin A and are stimulated by retinoic acid gene 8 (Stra8) (Bowles et al., 2006; Koubova et al., 2006), a key regulator of meiosis initiation. In foetal testicular germ cells, the expression of Stra8 is suppressed by FGF9 (Bowles et al., 2010), Nanos2 (Suzuki & Saga, 2008; Tsuda et al., 2003), DMRT1 (Matson et al., 2010) and Cyp26b1, which inactivates RA (Bowles et al., 2006; Koubova et al., 2006). However, it remains largely unknown how meiosis is specifically promoted and maintained in postnatal testis.

Family members of SRY (sex‐determining region Y)‐box (SOX) are transcription factors containing a highly conserved HMG (high mobility group)‐box for DNA‐binding (Chew & Gallo., 2009; Gubbay et al., 1990; Sekido & Lovell‐Badge, 2009). Over twenty different SOXs have been recognized in mammals. Growing evidence indicates that Sox genes play key roles in diverse embryonic and postnatal developmental processes, including cell fate decision, sex determination and differentiation (Kamachi & Kondoh, 2013; Sarkar & Hochedlinger K, 2013; Sinclair et al., 1990). SRY‐box 30 (Sox30), a relatively new SOX member, was first cloned from the mouse testis. For a long time, *Sox30* has been thought to exist only in mammals and its specific role remains unclear. In our previous study, we confirmed for the first time that *Sox30* is not only a gene that only exists in mammals, but also exists widely throughout the animal kingdom (Han et al., 2010), suggesting that this gene may be more important than previously considered. Recently, *SOX30* was first identified as a novel and key tumour suppressor in lung and ovarian cancers (Han et al., 2015, 2019; Han, Liu, et al., 2018; Han, Zhang, et al., 2018), and then, as well as in other types of cancers (Guo et al., 2017; Liu et al., 2018; Tao et al., 2019). Our latest study has revealed that epigenetic silencing of SOX30 is a key contributor to male infertility and may offer a therapeutic target for azoospermia in humans (Han et al., 2020). Based on the expression pattern in testis development, *Sox30* has been considered likely to be involved in spermatogenesis (Han et al., 2010, 2014; Osaki et al., 1999; Petit et al., 2015). Recent research has determined that *Sox30* is required for spermiogenesis in mice (Bai et al., 2018; Feng et al., 2017; Zhang et al., 2018). However, it remains unclear whether Sox30 is involved in germ‐cell meiosis of postnatal testis.

Our previous study has indicated that *Sox30* expression might be associated with germ‐cell meiosis in mouse testes (Han et al., 2014). Herein, we generated an inducible Sox30‐null mouse model and first revealed that Sox30‐null male mice showed arrested meiosis of spermatocyte and uncontrollable proliferation of Leydig cells, resulting in the complete absence of spermatozoa. At the molecular level, Sox30 dominates male germ‐cell meiosis via regulating RA‐signalling through direct transcriptional suppression of *Cyp26b1*, and via direct transcriptional activation of downstream targets *Rec8* and *Stra8*. Furthermore, Sox30 promotes male differentiation by regulating critical genes of sex differentiation (such as *Rspo1*, *Foxl2*, *Sox9* and *Ctnnb1*), especially by direct transcriptional suppression of *Ctnnb1*. The defects of germ‐cell differentiation and Leydig cell proliferation can be ameliorated in Sox30‐null mice after re‐expression of *Sox30*, which is indeed associated with regulation of *Cyp26b1*, *Stra8*, *Rec8* and *Ctnnb1*. This study identified *Sox30* as a key gatekeeper for male germ‐cell meiosis and differentiation, providing new insights into the meiosis of postnatal testis and male differentiation.

## 2 RESULTS

### 2.1 *Sox30*‐Null impairs testes exhibiting abnormal cell differentiation and proliferation

**FIGURE 1:**
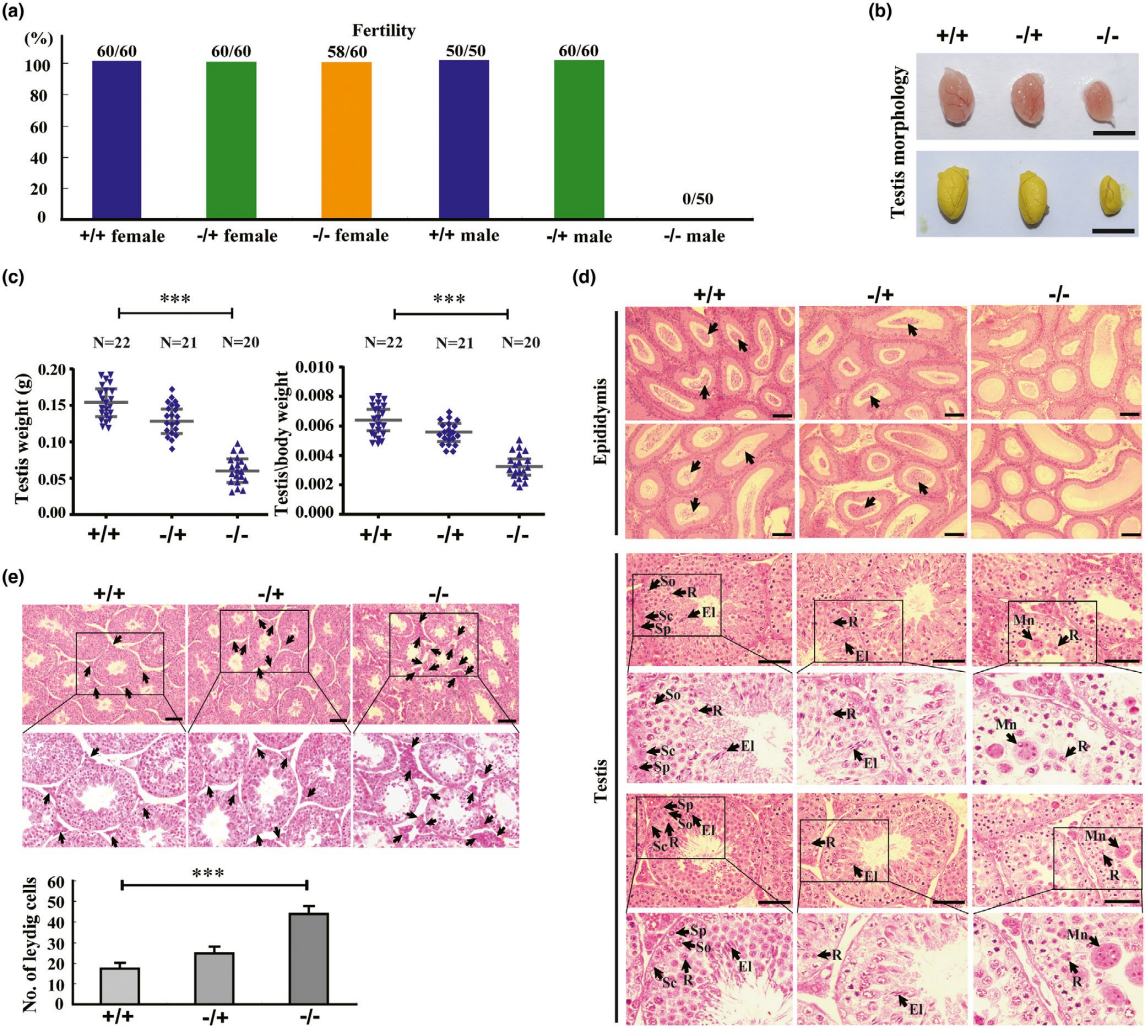
Sox30‐null specifically impairs male fertility and testis with abnormal cell differentiation and proliferation. (a) Fertility was analysed in different Sox30 genotypes of mice. “+/+” represents wild‐type mice (Sox30^+/+^), “‐/+” represents heterozygous mice (Sox30^−/+^) and “‐/‐” represents homozygous mice (Sox30^−/−^). (b) Testicular morphology was evaluated in Sox30^+/+^, Sox30^−/+^ and Sox30^−/−^ adult mice. “***” represents a p‐value less than 0.001. The scale bar is 0.5 cm. (c) The testis weight and testis weight/body weight were analysed in Sox30^+/+^, Sox30^−/+^ and Sox30^−/−^ adult mice. “***” represents a p‐value less than 0.001. (d) Haemotoxylin‐eosin staining of sections of epididymide and testis are shown in Sox30^+/+^ (n = 8), Sox30^−/+^ (n = 8) and Sox30^−/−^ (n = 8) mice at 4 months post‐partum. No spermatozoa in Sox30^−/−^ epididymides and complete absence of spermatozoa with few spermatids and some spermatocyte‐like cells in seminiferous tubules of Sox30^−/−^ testes can be observed. The arrows represent spermatozoa in epididymides. The arrows with “Sp” represent the spermatogonia, the arrows with “So” represent the spermatocytes, the arrows with “Sc” represent the Sertoli cells, the arrows with “R” represent the round spermatids, the arrows with “El” represent the elongated spermatids, and the arrows with “Mn” represent multi‐nucleated cells in testes. Scale bars are 50 µm. (e) Leydig cells in testes were compared between Sox30^+/+^ (n = 8), Sox30^−/+^ (n = 8) and Sox30^−/−^ (n = 8) adult (4 months) mice. The cell numbers were quantified based on an average of 5 random fields, and the results were normalized by the area and number of seminiferous tubules in the area. The arrows represent Leydig cells. “***” represents a p‐value less than 0.001. Scale bars are 50 µm

To determine the precise role of *Sox30*, we generated an inducible Sox30‐null mouse model (Figure S1). Homozygous (Sox30**^−/−^**) and heterozygous (Sox30**^−/+^**) mice developed normally and lived more than one year without an obvious difference in general physical appearance when compared with their wild‐type (Sox30**^+/+^**) littermates. Adult Sox30**^+/+^**, Sox30**^−/+^** and Sox30**^−/−^** female mice showed 100% (60/60), 100% (60/60) and 96.67% (58/60) fertility, respectively (Figure 1a). Both adult Sox30**^+/+^** and Sox30**^−/+^** male mice showed 100% (50/50) and 100% (60/60) fertility, respectively (Figure 1a). However, the adult Sox30**^−/−^** male mice demonstrated complete infertility (fertile in 0/50, Figure 1a). Correspondingly, Sox30**^−/−^**, Sox30**^−/+^** and Sox30**^+/+^** female mice demonstrated similar ovaries, but Sox30**^−/−^** male mice showed significantly small testes when compared with Sox30**^+/+^** or Sox30**^−/+^** mice (Figure 1b,c; Figure S2A,B).

To determine the causes of infertile and testis impairment, we observed the gametes in epididymides and testes of Sox30**^−/−^** mice by histological examination. No spermatozoa were observed in epididymides of Sox30**^−/−^** adult mice (Figure 1d). A complete absence of spermatozoa and elongated spermatids, as well as reduced round spermatids, was observed in seminiferous tubules of testes from Sox30**^−/−^** adult mice (Figure 1d). The phenotypes for no spermatozoa in epididymides and complete absence of spermatozoa and elongated spermatids in testes of Sox30**^−/−^** adult mice were further confirmed by analysing semen with Sperm Class Analyzer (SCA) in epididymides and testes micro‐morphology examination using an electron‐microscopy (Figure S3A,B). Furthermore, the number of Leydig cells was significantly increased in testes of Sox30**^−/−^** adult mice (Figure 1e). These results revealed that loss of *Sox30* in mice specifically impairs male fertile and testis development by affecting germ‐cell differentiation and somatic Leydig cell proliferation.

### 2.2 The impairments of testes become more severe in Sox30‐null aged mice

**FIGURE 2:**
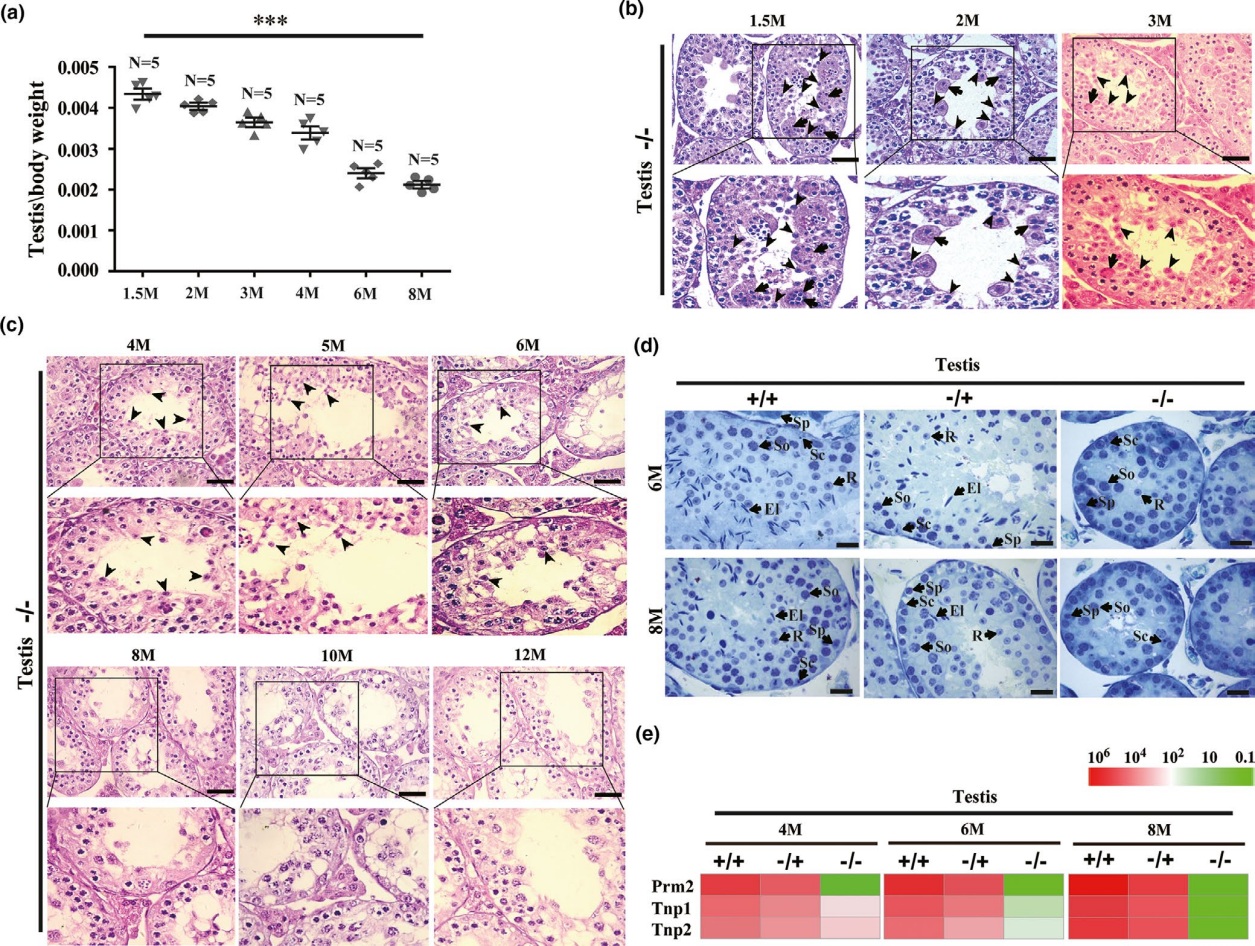
Testicular impairments become increasingly severe in ageing Sox30^−/−^ mice. (a) The testis/body weight was analysed in Sox30^+/+^, Sox30^−/+^ and Sox30^−/−^ mice at 1.5 months (1.5 M), 2 months (2 M), 3 months (3 M), 4 months (4 M), 6 months (6 M) and 8 months (8 M) post‐partum. “***” represents a p‐value less than 0.001. (b, c) Histopathology of testes was compared among Sox30^−/−^ mice at 1.5 months (1.5 M), 2 months (2 M), 3 months (3 M), 4 months (4 M), 5 months (5 M), 6 months (6 M), 8 months (8 M), 10 months (10 M) and 12 months (12 M) post‐partum. Round spermatids are still observed in Sox30^−/−^ testes at and before 6 months post‐partum. The complete absence of round spermatids and a significant reduction in spermatocytes can be observed in Sox30^−/−^ testes after 6 months post‐partum. The arrows with long tails represent multi‐nucleated cells, and the arrows with short tails represent round spermatids. The scale bars are 50 µm. (d) Semithin histological sections stained with toluidine blue were performed in testes of Sox30^+/+^, Sox30^−/+^ and Sox30^−/−^ mice at 6 months (6 M) and 8 months (8 M) post‐partum. The arrows with “Sp” represent the spermatogonia, the arrows with “So” represent the spermatocytes, the arrows with “Sc” represent the Sertoli cells, the arrows with “R” represent the round spermatids and the arrows with “El” represent the elongated spermatids. Scale bars are 50 µm. (E) Heatmap of relative expression was analysed for the markers of round spermatids and elongated spermatids in testes of Sox30^+/+^, Sox30^−/+^ and Sox30^−/−^ mice at 4 months (4 M), 6 months (6 M) and 8 months (8 M) post‐partum by RT‐qPCR

Smaller testes were observed in Sox30**^−/−^** male mice when compared with Sox30**^+/+^** and Sox30**^−/+^** mice. Interestingly, the loss of Sox30^−/−^ testicular weight was more severe as mice aged from 1.5 months to 8 months post‐partum (Figure 2a). Pathological examination showed that complete absence of spermatozoa and elongated spermatids was observed in testes of Sox30**^−/−^** mice at all nine developmental stages (1.5, 2, 3, 4, 5, 6, 8, 10 and 12 months post‐partum), and round spermatids were observed in testes of Sox30**^−/−^** mice at and before 6 months post‐partum (Figure 2b,c). Evidently, round spermatids in testes of Sox30**^−/−^** mice decreased in continuity from 1.5 months to 6 months post‐partum, and completely disappeared in testes of Sox30**^−/−^** mice at and after 8 months post‐partum (Figure 2b,c). The above results were further confirmed in independent samples by semithin histological section and expression analysis of spermatid markers: Tnp1, Tnp2 and Prm2 (Tnp1 and Tnp2 are spermatid markers, and Prm2 is an elongated spermatid marker) (Figure 2d,e). These data revealed that impairments of Sox30‐null on testes become more severe as mice aged, suggesting a major impairment in prophase of germ‐cell differentiation occurred at least before round spermatid formation.

### 2.3 The germ cells are arrested at early meiotic prophase in Sox30‐null testes

**FIGURE 3:**
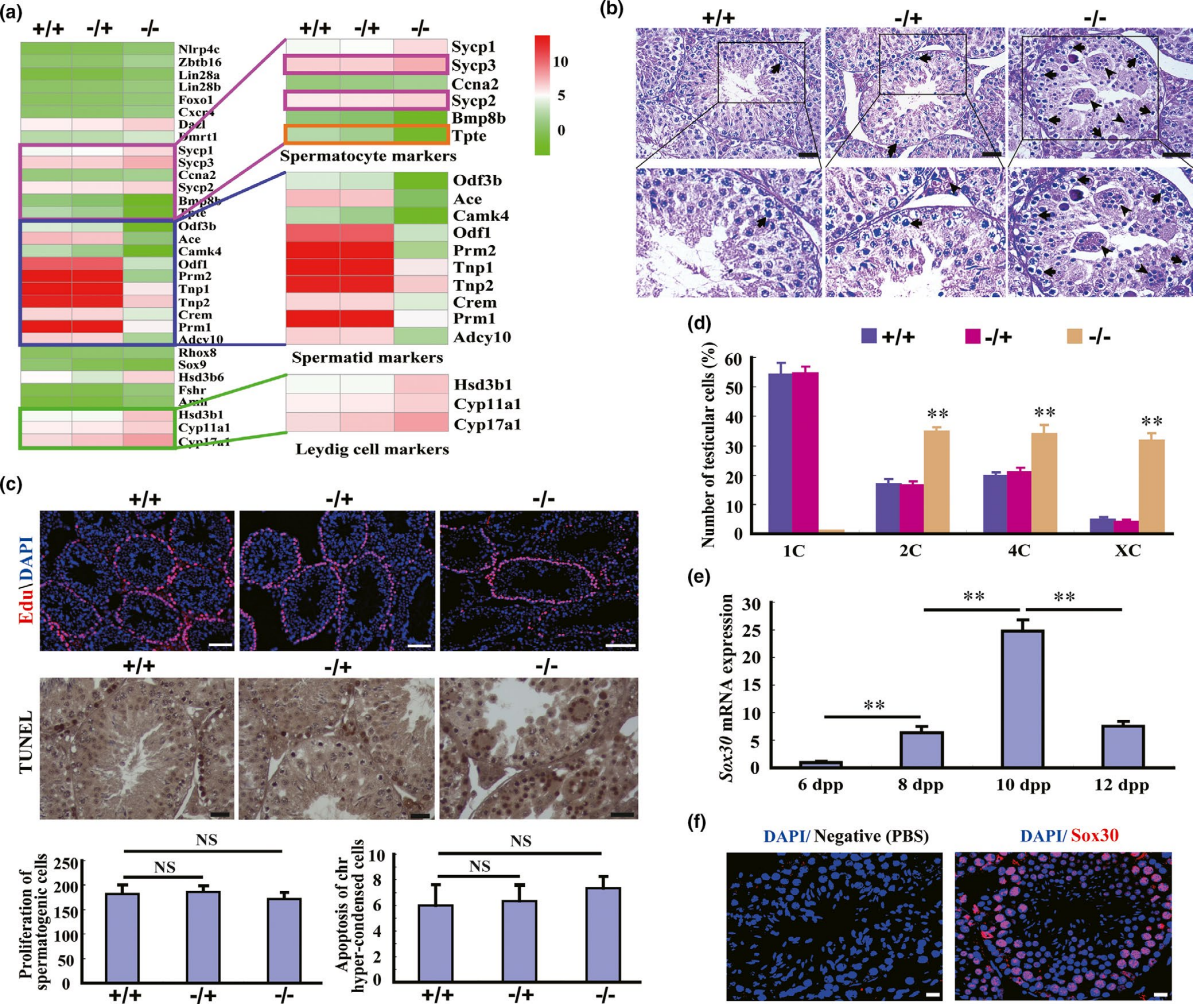
Germ‐cell meiosis and differentiation is abnormal in testes of Sox30‐null mice. (a) The different expressions of markers for spermatocytes, spermatids and Leydig cells were observed in testes of Sox30**^+/+^**, Sox30^−/+^ and Sox30**^−/−^** mice by RNA sequencing. (b) Haematoxylin‐eosin staining was performed in the testes of Sox30**^+/+^**, Sox30^−/+^ and Sox30**^−/−^** mice. Some multi‐nucleated spermatogenic cells are present in the testes of Sox30**^−/−^** adult (2 months) mice. The arrows with long tails indicate spermatocytes with condensed chromatin, and the arrows with short tails represent multi‐nucleated cells. Scale bars are 50 µm. (c) Spermatogenic cell proliferation and apoptosis were measured by 5‐ethynyl‐2’‐deoxyuridine (EdU) and TUNEL assays in adult (2 months) testes. The cell numbers were quantified based on an average of 5 random fields and was normalized by the area and number of seminiferous tubules in the area. The mice were intraperitoneally injected with 100 µg of EdU in phosphate‐buffered saline (PBS), and spermatogenic cell proliferation assays were performed after incorporation for 72 h. TUNEL positive apoptotic cells are stained brown. The “NS (no significance)” represents a p‐value more than 0.05. Scale bars represent 50 µm. (d) The DNA content of testicular cells was determined by flow cytometric analyses in testes of Sox30**^+/+^**, Sox30^−/+^ and Sox30**^−/−^** adult (4 months) mice. The 1C (one copy of chromosome) peak was determined by comparison with sperms of the epididymis. “**” represents a p‐value less than 0.01. (e) The expression of *Sox30* was evaluated in testes of 6 days post‐partum (dpp), 8 dpp, 10 dpp and 12 dpp old wild‐type mice. *Gapdh* was used as an internal control. “**” represents a p‐value less than 0.01. (f) Sox30 expression was analysed in the testes of wild‐type adult (4 months) mice by immunofluorescence staining. Sox30 is mainly expressed in spermatocytes and marginally expressed in round spermatids. As a negative control, a primary antibody was replaced with PBS. Scale bars are 20 µm

On analysing transcriptomes of testes from Sox30^+/+^, Sox30**^−/+^** and Sox30**^−/−^** mice using RNA sequencing, numerous spermatid markers such as Tnp1, Tnp2, Prm2 and Prm1 were significantly reduced and Leydig cell markers such as Hsd3b1 and Cyp17a1 were elevated in testis of Sox30**^−/−^** mice (Figure 3a), which further confirmed the above results of pathological and semithin histological examination. Notably, the markers of primary spermatocytes such as Sycp2 and Sycp3 were significantly enhanced, whereas markers of secondary spermatocytes such as Tpte were markedly reduced (Figure 3a). Then, we focused on comparing the nuclear morphology of germ cells in different genotype testes and observed several multi‐nucleated spermatogenic cells and chromosome hyper‐condensed spermatogenic cells in testes of Sox30**^−/−^** mice (Figure 3b), indicating abnormal mitosis or meiosis and apoptosis or characterising early meiotic prophase, respectively. To determine the possible causes of multi‐nucleated spermatogenic cells and chromosome hyper‐condensed spermatogenic cells, we first tested spermatogenic cell proliferation by performing a 5‐ethynyl‐2’‐deoxyuridine (EdU) assay and observed no significant change in spermatogenic cell proliferation among Sox30**^+/+^**, Sox30**^+/−^** and Sox30**^−/−^** mice, suggesting that the mitosis of germ cells was normal (Figure 3c). Next, we evaluated the apoptotic events of these cells by performing a terminal deoxynucleotidyl transferase‐mediated dUTP nick end labelling (TUNEL) assay and failed to observe extensive apoptosis of chromosome hyper‐condensed spermatogenic cells (Figure 3c). Furthermore, DNA content analyses of testicular cells by flow cytometry showed few cells harbouring one copy of a chromosome (1C, different steps of spermatids and spermatozoa), an increase in 2C cells (G1 phase spermatogonia, secondary spermatocytes, somatic Sertoli cells and somatic Leydig cells), an accumulation of 4C cells (primary spermatocytes and G2 phase spermatogonia) and XC cells (multi‐nucleated spermatogenic cells, X > 4) in the testes of Sox30**^−/−^** mice (Figure 3d). These results indicated that testicular germ cells are probably arrested at early meiotic prophase in Sox30**^−/−^** mice.

To further confirm the above hypothesis and determine the arrest phase, we then examined *Sox30* expression in postnatal testes from 6 to 12 dpp (a key time period of meiosis) old wild‐type C57BL/6 mice. During this period, *Sox30* expression started increasing in testes of mice at 8 dpp and peaked at 10 dpp, which is a critical time point for spermatocyte meiosis (Figure 3e). In wild‐type C57BL/6 mice, the first spermatogenic meiosis occurs at 8 dpp, and then progresses to leptotene, zygotene and pachytene stages at 10, 12 and 14 dpp, respectively. Moreover, Sox30 expression is mainly restricted to spermatocytes (Figure 3f). These results revealed that the arrest of germ‐cell meiosis is associated with leptotene or zygotene stage spermatocytes in Sox30**^−/−^** mice.

### 2.4 The germ‐cell meiosis is arrested at the zygotene stage in testes of Sox30‐null mice

**FIGURE 4:**
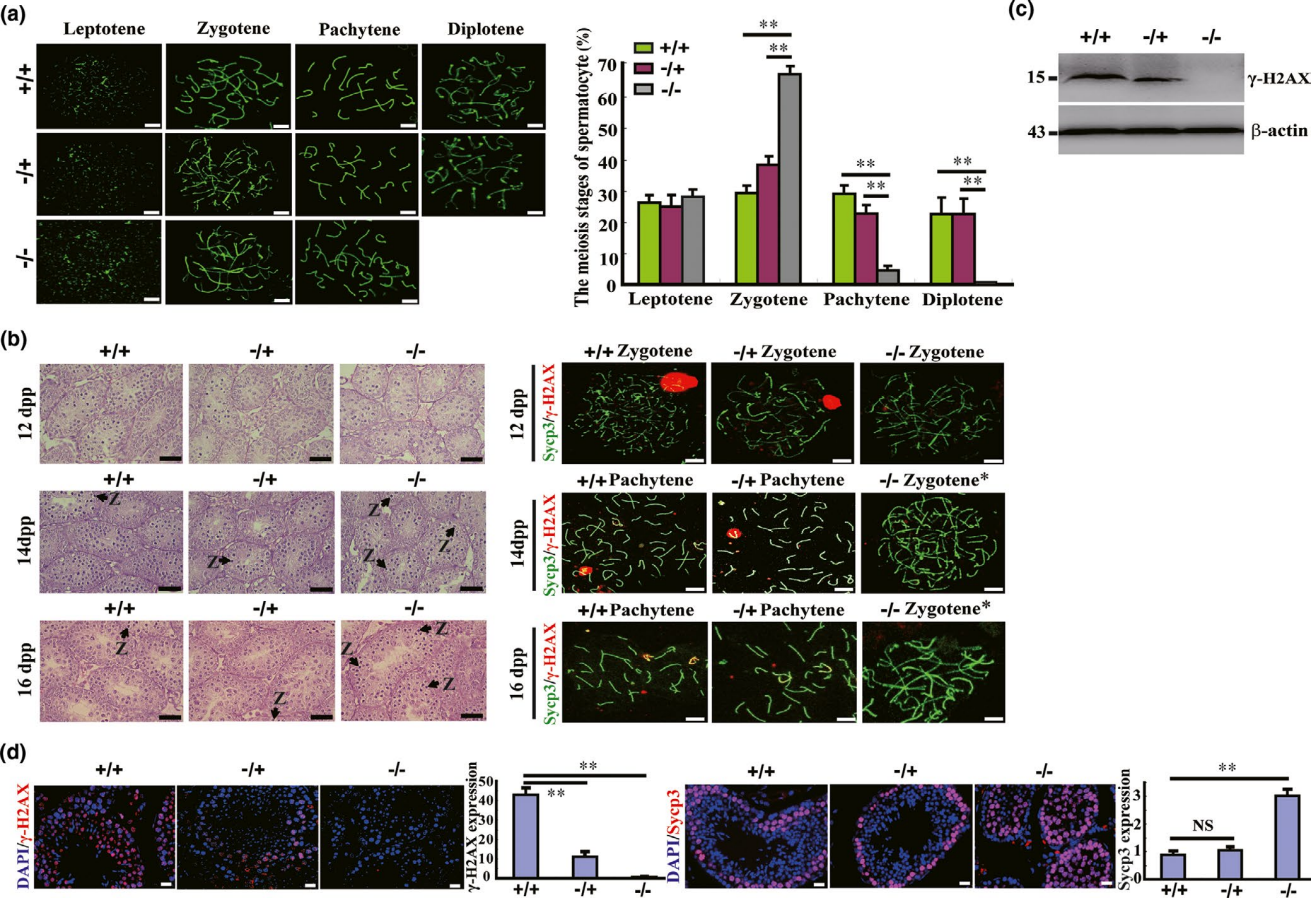
Germ‐cell meiosis and differentiation is arrested at the zygotene stage in testes of Sox30‐null mice. (a) Quantification of spermatocytes at different meiotic stages was detected by chromosome spreads staining of Sycp3 in testes of Sox30^+/+^ (n = 50), Sox30^−/+^ (n = 50) and Sox30^−/−^ (n = 50) adult (3 months) mice. “**” represents a p‐value less than 0.01. Scale bars are 20 µm. (b) Haematoxylin‐eosin (H&E) staining and chromosome spread staining of Sycp3 and γ‐H2AX for spermatocytes were analysed in testes of Sox30^+/+^, Sox30^−/+^ and Sox30^−/−^ mice at 12 days post‐partum (dpp), 14dpp and 16dpp stages. The arrows in the H&E staining of histological sections indicate spermatocytes with zygotene (Z) nuclear morphologies. Scale bars are 50 µm. “*” in histological sections of chromosome spreads staining of Sycp3 and γ‐H2AX represent spermatocyte arrest at zygotene stage. Scale bars are 20 µm (c, d). The expression of DSB marker γ‐H2AX and Sycp3 was determined by western blotting or immunofluorescence staining in testes of Sox30^+/+^, Sox30^−/+^ and Sox30^−/−^ adult (3 months) mice. Relative expression was quantified based on mean grey value of an average of 5 random fields. “**” represents a p‐value less than 0.01. “NS” represents a p‐value more than 0.05

To determine the precise phase of meiotic spermatocyte arrest in Sox30**^−/−^** mice, chromosome spread assays were performed. In the testes of Sox30**^−/−^** mice, leptotene spermatocytes were not altered, while zygotene spermatocytes were accumulated (67.01% [321/497] in Sox30**^−/−^** mice vs. 39.23% [193/492] in Sox30**^−/+^** mice vs. 30.42% [174/572] in Sox30**^+/+^** mice) and pachytene and post‐pachytene spermatocytes were markedly reduced (Figure 4a). To validate the defect of zygotene‐to‐pachytene transition in Sox30**^−/−^** mice, we further examined the meiotic process in testes from 10, 12, 14 and 16 dpp old Sox30**^+/+^**, Sox30**^−/+^** and Sox30**^−/−^** mice by performing histological examination and chromosome spread assays. Zygotene cell accumulation was also observed in the testes of Sox30**^−/−^** mice at 14 and 16 dpp (Figure 4B; Figure S4A,B). Consistent with the arrest of meiosis at zygotene (start at 12 dpp), the difference in testis weight presented no statistical significance among Sox30**^+/+^**, Sox30**^−/+^** and Sox30**^−/−^** mice at 8, 10 and 12 dpp, whereas testis weight was lower in Sox30**^−/−^** mice than in Sox30**^+/+^** and Sox30**^+/−^** mice at 14 and 16 dpp (*p* < 0.05, Figure S4C). In zygotene, the key event is that homologous chromosomes start to pair and synapse, initiating the assembly of the synaptonemal complex (Lee & Hirano, 2011). In mice, homologous chromosomes start to synapse using a DNA double‐strand break (DSB)‐dependent mechanism (Cahoon & Hawley, 2016). Thus, to further confirm the arrest of meiosis at zygotene in Sox30**^−/−^** mice, we determined the expression of DSB marker γ‐H2A.X, and found that it was poorly detectable in the testes of Sox30**^−/−^** mice (Figure 4B‐D). Additionally, immunofluorescence with Sycp3 (a marker of primary spermatocyte) confirmed that spermatocytes were indeed increased in testes of Sox30**^−/−^** mice (Figure 4D). These data demonstrated that the zygotene‐to‐pachytene transition of germ‐cell meiosis was defective in Sox30**^−/−^** testes.

### 2.5 The meiotic defect of Sox30‐null mice is associated with critical regulators of meiosis and sex differentiation

**FIGURE 5:**
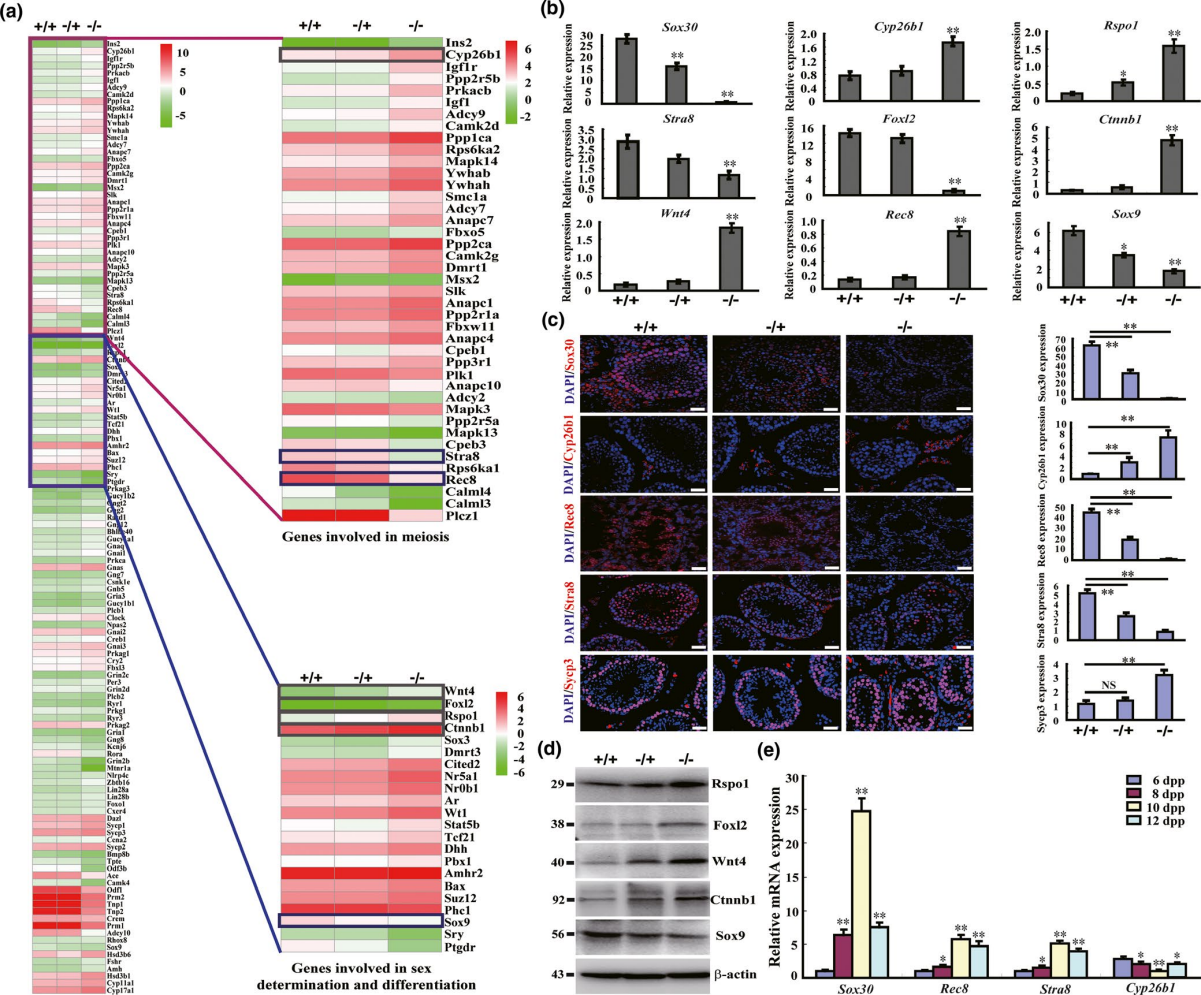
Sox30 is associated with critical regulators of meiosis and sex differentiation. (a) Hierarchical clustering of differentially expressed genes (≥1.5 fold) was analysed in testes of Sox30^+/+^, Sox30^−/+^ and Sox30^−/−^ mice. The critical regulators of germ‐cell meiosis, and sex differentiation were identified, including downregulation of *Stra8*, *Rec8* and *Sox9*, and upregulation of *Cyp26b1*, *Foxl2*, *Wnt4*, *Ctnnb1*, *Rspo1* and *Wt1*. (b) Expression levels of *Sox30*, *Cyp26b1*, *Stra8*, *Rec8*, *Sox9*, *Foxl2*, *Wnt4*, *Ctnnb1* and *Rspo1* were determined by RT‐qPCR analyses in testes of Sox30^+/+^, Sox30^−/+^ and Sox30^−/−^ mice. “**” represents a p‐value less than 0.01. “*” represents a p‐value less than 0.05. β‐actin was used as an internal control. (c) The expression levels of Sox30, Cyp26b1, Rec8, Stra8 and Sycp3 were evaluated by immunofluorescence staining in testes of Sox30^+/+^, Sox30^−/+^ and Sox30^−/−^ adult (3 months) mice. Relative expression was quantified based on mean grey value of an average of 5 random fields. “**” represents a p‐value less than 0.01. “NS” represents a p‐value more than 0.05. Scale bars are 20 µm. (d) The protein expressions of Sox9, Foxl2, Wnt4, Ctnnb1 and Rspo1 was evaluated by western blotting in testes of Sox30^+/+^, Sox30^−/+^ and Sox30^−/−^ adult (3 months) mice. (e) RT‐qPCR analyses of expression levels of *Sox30*, *Rec8*, *Stra8* and *Cyp26b1* were performed in testes of 6 (n = 6), 8 (n = 6), 10 (n = 6) and 12 (n = 6) days post‐partum (dpp) old wild‐type mice. *Gapdh* was used as an internal control. “**” represents a p‐value less than 0.01. “*” represents a p‐value less than 0.05

To address the mechanism underlying the arrest of meiosis, we re‐analysed the transcriptomes of testes from Sox30^+/+^, Sox30**^−/+^** and Sox30**^−/−^** mice. The expression of several critical regulators for meiosis and sex differentiation was affected by *Sox30* deletion, including *Stra8*, *Rec8* and *Sox9* were downregulated, and *Cyp26b1*, *Foxl2*, *Wnt4*, *Ctnnb1 (β*‐*catenin)*, *Rspo1* and *Wt1* were upregulated (Figure 5A). The expression changes of these genes were confirmed in the testes of Sox30**^+/+^**, Sox30**^−/+^** and Sox30**^−/−^** mice by quantitative reverse transcription PCR (RT‐qPCR), immunofluorescence staining and western blotting (Figure 5B‐D; Figure S5A,B). Furthermore, the expression of *Sox30* was positively correlated with *Stra8* and *Rec8*, while negatively associated with *Cyp26b1* in the testes of wild‐type mice at 6, 8, 10 and 12 dpp (Figure 5E). These data revealed that *Sox30* probably regulates the key meiotic genes, *Cyp26b1*, *Stra8* and *Rec8*, as well as critical genes of sex differentiation, *Sox9*, *Foxl2*, *Wnt4*, *Ctnnb1* and *Rspo1*.

### 2.6 Sox30 regulates *Cyp26b1*, *Stra8*, *Rec8*, and *Ctnnb1* by direct binding to their promoters

**FIGURE 6:**
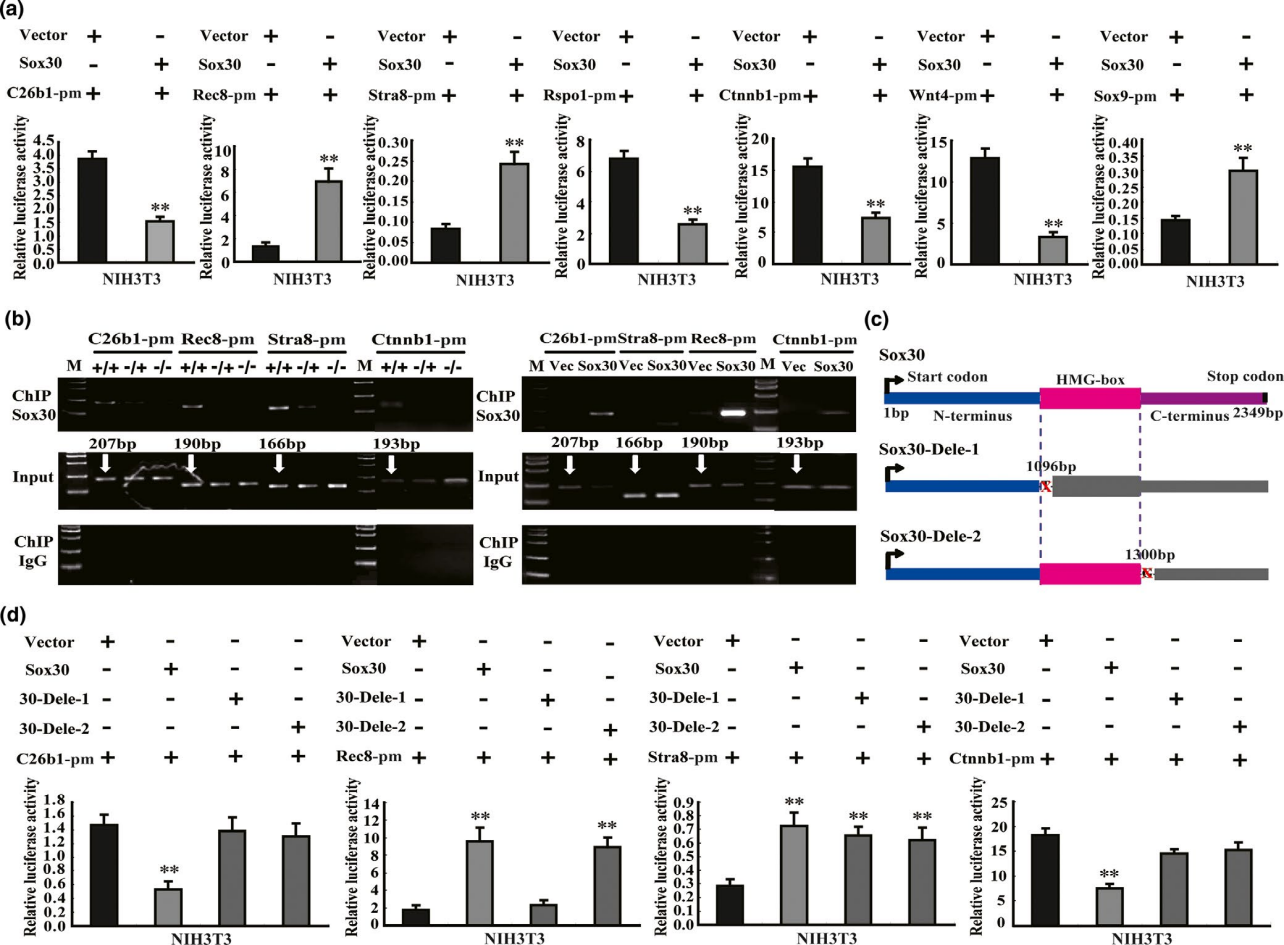
Sox30 regulates *Stra8*, *Rec8*, *Cyp26b1* and *Ctnnb1* by direct binding to their promoters. (a) The inhibition and activation of *Cyp26b1*, *Rec8*, *Stra8*, *Sox9*, *Wnt4*, *Ctnnb1* and *Rspo1* promoters were determined using luciferase reporter assays in NIH3 T3 cells. The results were normalized with internal controls and are presented as average with the standard error of the mean (SEM) from three experiments. C26b1‐pm represents *Cyp26b1* promoter, Rec8‐pm represents *Rec8* promoter, Stra8‐pm represents *Stra8* promoter, Sox9‐pm represents *Sox9* promoter, Wnt4‐pm represents *Wnt4* promoter, Ctnnb1‐pm represents *Ctnnb1* promoter and Rspo1‐pm represents *Rspo1* promoter. “**” represents a p‐value less than 0.01. (b) Chromatin immunoprecipitation analyses of Sox30 binding to the promoters of *Cyp26b1*, *Rec8*, *Stra8* and *Ctnnb1* were performed in testes of Sox30^+/+^, Sox30^−/+^ and Sox30^−/−^ adult (3 months) mice or transfected NIH3 T3 cells. The immunoprecipitates were analysed by RT‐PCR. C26b1‐pm represents *Cyp26b1* promoter, Rec8‐pm represents *Rec8* promoter, Stra8‐pm represents *Stra8* promoter and Ctnnb1‐pm represents *Ctnnb1* promoter. The “Vec” represents an empty vector. “M” represents DNA marker. (c) The vectors without HMG‐box and C‐terminal domain or without only C‐terminal domain of Sox30 were constructed. Sox30‐Dele‐1 and Sox30‐Dele‐2 represent the plasmid without HMG‐box and C‐terminal regions, and the plasmid without C‐terminal region, respectively. (d) The effect of Sox30 with or without HMG‐box and/or C‐terminal domains on *Cyp26b1*, *Rec8*, *Stra8* and *Ctnnb1* promoter activities was analysed. C26b1‐pm represents *Cyp26b1* promoter, Rec8‐pm represents *Rec8* promoter, Stra8‐pm represents *Stra8* promoter and Ctnnb1‐pm represents *Ctnnb1* promoter. “**” represents a p‐value less than 0.01

To elucidate the molecular mechanism underlying Sox30 regulation of *Cyp26b1*, *Rec8*, *Stra8*, *Sox9*, *Foxl2*, *Wnt4*, *Ctnnb1* and *Rspo1*, we analysed whether it could regulate these targets at the transcriptional level as *Sox30* acts as a transcription factor. Bioinformatic analyses revealed that *Cyp26b1*, *Rec8*, *Stra8*, *Sox9*, *Foxl2* and *Ctnnb1* promoters contain Sox30 binding sites (Table S1). The full‐length promoters of *Cyp26b1*, *Rec8*, *Stra8*, *Wnt4*, *Sox9*, *Rspo1*, *Foxl2* and *Ctnnb1* were then cloned into a pGL3‐basic luciferase reporter vector and co‐transfected with pIRES2‐EGFP‐Sox30 or pIRES2‐EGFP‐vector into NIH3 T3 and/or HEK293 cells (both cell lines show low Sox30 expression originally), and luciferase reporter assays were performed. The experimental results indicated that Sox30 significantly attenuated activities of *Cyp26b1* (−1891 to +144 bp), *Wnt4* (−2000 to +100 bp), *Rspo1* (−2000 to +250 bp) and *Ctnnb1* (−2000 to +200 bp) promoters, and strengthened activities of *Rec8* (−1800 to +136 bp), *Stra8* (−1900 to +100 bp) and *Sox9* (−2000 to +400 bp) promoters, not affecting the activity of *Foxl2* (−2000 to +150 bp) promoter (Figure 6A; Figure S6A). As there were only several potential binding sites for Sox30 in *Cyp26b1*, *Rec8*, *Stra8*, *Sox9* and *Ctnnb1* promoters, we designed pairs of primers according to the potential binding sites, respectively, and performed chromatin immunoprecipitation (ChIP)‐PCR assays in testis tissues of Sox30**^+/+^**, Sox30**^−/+^** and Sox30**^−/−^** mice, and transfected NIH3 T3/HEK293 cells (as no binding sites of Sox30 were found in *Rspo1* and *Wnt4* promoters and Sox30 did not affect the activity of *Foxl2* promoter, the ChIP‐PCR of *Rspo1*, *Wnt4* and *Foxl2* was not performed). ChIP‐PCR data revealed that Sox30 regulated the expression of *Cyp26b1*, *Stra8*, *Rec8* and *Ctnnb1* by direct binding to their promoters (Figure 6B). These findings demonstrated that *Cyp26b1*, *Rec8*, *Stra8* and *Ctnnb1* are direct downstream targets of Sox30.

### 2.7 Different domains of Sox30 are required for affecting activities of *Cyp26b1*, *Rec8*, *Stra8*, and *Ctnnb1* promoters

To determine the potential key domain or whether the HMG‐box (DNA‐binding domain) is required for Sox30 affecting activities of *Cyp26b1*, *Rec8*, *Stra8* and *Ctnnb1* promoters, deletion of the HMG‐box domain (366‐433AA) and carboxyl‐terminal (C‐terminus) region (434‐783AA) or deletion of only the C‐terminus region were generated by site‐directed mutagenesis (Figure 6C). Sox30 failed to attenuate *Cyp26b1* and *Ctnnb1* promoter activities when the HMG‐box domain and C‐terminus region were both deleted; furthermore, it failed to attenuate *Cyp26b1* and *Ctnnb1* promoter activities when only the C‐terminus region was deleted (Figure 6D; Figure S6B). Sox30 failed to activate *Rec8* promoter activity when the HMG‐box domain and C‐terminus region were both deleted, whereas it still activated *Rec8* promoter activity when only the C‐terminus region was deleted (Figure 6D; Figure S6C). Sox30 activated *Stra8* promoter activity when the HMG‐box domain and C‐terminus region were both deleted or when only the C‐terminus region was deleted (Figure 6D; Figure S6C). These data suggested that the C terminus of Sox30 is required for attenuating *Cyp26b1* and *Ctnnb1* promoter activity, and the HMG‐box of Sox30 is required for stimulating *Rec8* promoter activity, while the amino‐terminal (N‐terminus) region (1‐365AA) of Sox30 may be required for activating *Stra8* promoter activity, as neither the HMG‐box nor the C terminus of Sox30 was required.

### 2.8 RA level is reduced in Sox30‐null testes owing to increased degradation rather than decreased production

As Cyp26b1 oxidizes RA to an inactive metabolite, we then determined the RA level in testes of Sox30**^+/+^**, Sox30**^−/+^** and Sox30**^−/−^** adult mice. It was observed that the RA levels were markedly decreased in the testes of Sox30**^−/−^** mice when compared with the testes of Sox30**^+/+^** mice, with a dose‐response relationship in testes from Sox30**^+/+^**, Sox30**^−/+^**, to Sox30**^−/−^** mice (Figure S7A). To exclude the possibility that decreased RA levels were due to the production rather than degradation, we assessed the expression of aldehyde dehydrogenase family 1 (ALDH1) and (ALDH2), two major enzymes responsible for RA production, and observed that ALDH1 and ALDH2 appeared to be minimally affected in testes of Sox30**^−/−^** mice (Figure S7B,C). Whereas Cyp26b1 was potently upregulated in testes of Sox30**^−/−^** mice, and a dose‐response relationship was observed for Cyp26b1 expression in testes from Sox30**^+/+^**, Sox30**^−/+^**, to Sox30**^−/−^** mice (Figure S7B,C). These data suggested that Sox30 can reduce RA degradation by inhibiting Cyp26b1 rather than increasing RA production by affecting ALDH1 and ALDH2.

### 2.9 *Sox30* is necessary and sufficient for germ‐cell meiosis and differentiation that associated with *Cyp26b1*, *Stra8*, *Rec8*, and *Ctnnb1*

**FIGURE 7:**
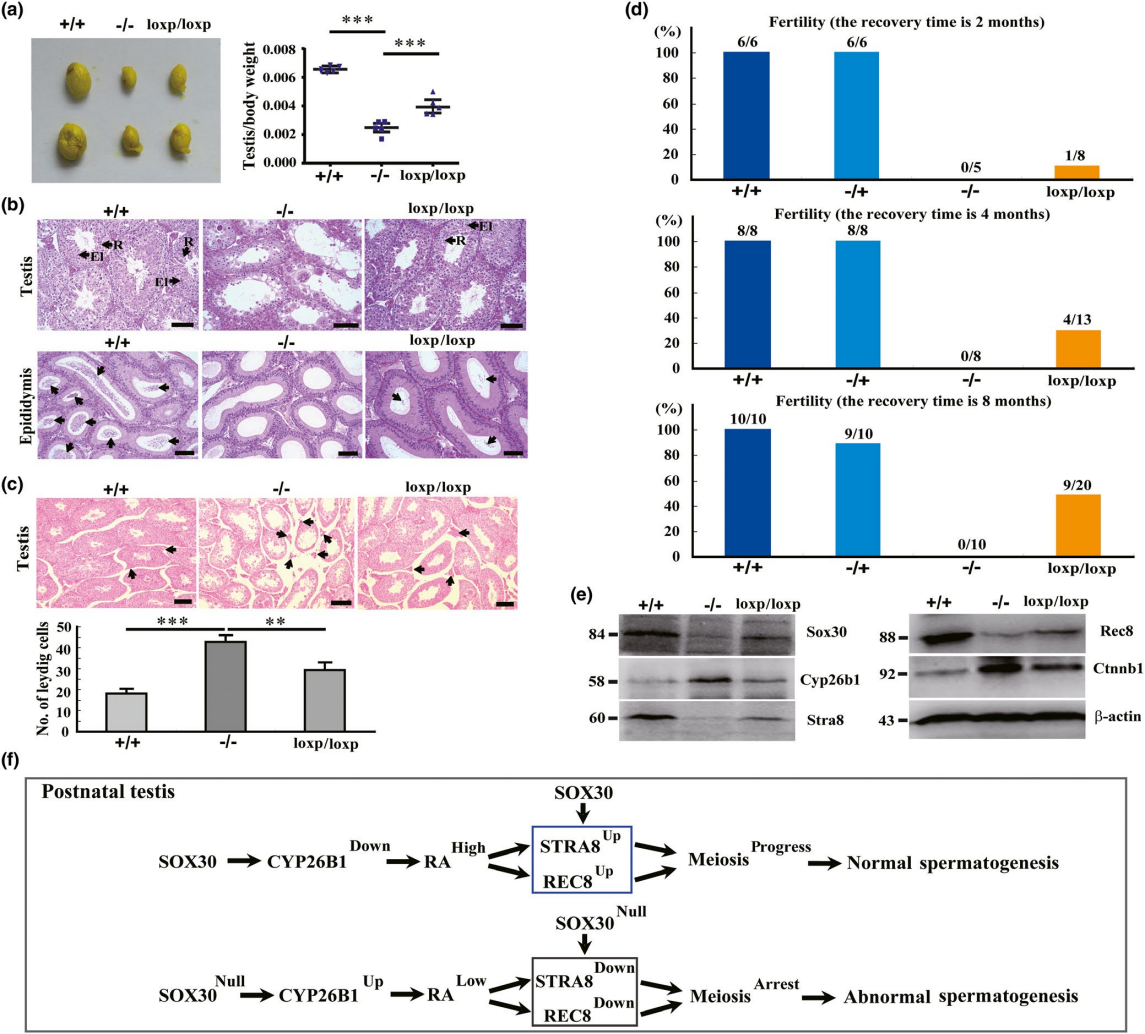
Sox30 is required for testis development and germ‐cell meiosis via regulation of *Cyp26b1*, *Stra8*, *Rec8*, and *Ctnnb1*. (a) The morphology and testis/body weight were analysed in Sox30^+/+^ (n = 5), Sox30^−/−^ (n = 5), and Sox30^loxp/loxp^ (n = 5) mice. +/+, Sox30^+/+^ mice injected with Tam; ‐/‐, Sox30^−/−^ mice injected with solvent; loxp/loxp, Sox30^−/−^ mice injected with Tam. “***” represents a p‐value less than 0.001. (b) H&E staining of the testes and epididymides was performed in Sox30^+/+^, Sox30^−/−^ and Sox30 re‐expressed Sox30^−/−^ (Sox30^loxp/loxp^) mice (the recovery time is 4 months). Numerous spermatids and spermatozoa are observed in the testes and several many spermatozoa are detected in the epididymides of Sox30^+/+^ mice (n = 5) after Tam injection, but are not observed in the testes and epididymides of Sox30^−/−^ (n = 5) mice after solvent injection. A considerable amount of spermatids and spermatozoa are detected in the testes of Sox30^loxp/loxp^ mice, and some spermatozoa can be found in the epididymides of these Sox30^loxp/loxp^ mice (n = 5). The arrows represent spermatozoa in epididymides. The arrows with “R” represent the round spermatids, and the arrows with “El” represent the elongated spermatids in testes. Scale bars are 100 µm (c). Leydig cells were quantified and compared in the testes of Sox30^+/+^, Sox30^−/−^ and Sox30^loxp/loxp^ mice. The cell numbers were quantified based on an average of 5 random fields, and the results were normalized by the area and number of seminiferous tubules in the area. The arrows represent Leydig cells. “***” represents a p‐value less than 0.001. “**” represents a p‐value less than 0.01. Scale bars are 50 µm. (d) Fertility was analysed in Sox30^−/−^ mice after re‐expression of Sox30 at 2, 4 and 8 months. +/+, Sox30^+/+^ mice injected with Tam; ‐/+, Sox30^−/+^ mice injected with Tam; ‐/‐, Sox30^−/−^ mice injected with solvent; loxp/loxp, Sox30^−/−^ mice injected with Tam. (e) Expression levels of Sox30, Cyp26b1, Stra8, Rec8 and Ctnnb1 were analysed by western blotting in the testes of Sox30^+/+^, Sox30^−/−^ and Sox30^loxp/loxp^ mice. β‐actin was used as an internal control. (f) A schematic illustration of Sox30 regulation of germ‐cell meiosis is shown. In postnatal testes, following Sox30 expression, Cyp26b1 expression is inhibited, and the retinoic acid (RA) level is increased, with decreased catabolism by Cyp26b1 promoting Stra8 and Rec8 expression or Sox30 directly promotes Stra8 and Rec8 expression; meiosis is in progress and spermatogenesis is normal. On silencing Sox30 expression, Cyp26b1 is highly expressed without inhibition of Sox30, the RA level is decreased, with increased catabolism by Cyp26b1 leading to downregulation of Stra8 and Rec8 expression or Sox30‐null directly results in downregulation of Stra8 and Rec8 expression; meiosis is arrested, and spermatogenesis is abnormal

To determine the recovery of germ‐cell meiosis and differentiation after re‐expression of *Sox30* in male Sox30**^−/−^** mice, we restored Sox30 expression in adult Sox30^−/−^ mice (Sox30^loxp/loxp^) by deleting the floxed cassette using tamoxifen (Tam), which can activate the ER‐Cre and evaluated the changes in testis size, germ‐cell differentiation, Leydig cell proliferation and male fertility. The testis size and weight of Sox30^loxp/loxp^ mice were restored when compared with those of Sox30**^−/−^** mice injected with the solvent (Figure 7A; Figure S8A). Histological data showed the presence of spermatids and spermatozoa in numerous seminiferous tubules of testes, as well as spermatozoa in epididymides from Sox30^loxp/loxp^ mice (Figure 7B). Furthermore, the increased Leydig cells were also reinhibited after restoring *Sox30* expression in the testes of Sox30^loxp/loxp^ mice (Figure 7C). The fertility analysis data revealed that the male Sox30^loxp/loxp^ mice (Sox30**^−/−^** mice injected with Tam) were actual fertile after restoring *Sox30* expression (Figure 7D). Moreover, this restored fertility appeared to improve further with increasing recovery time. At the recovery time of 2 months, 12.5% (1/8) of male Sox30^loxp/loxp^ mice were fertile, while 100% (6/6) of both male Sox30^+/+^ and Sox30^−/+^ mice injected with Tam were fertile; male Sox30**^−/−^** mice injected with solvent were fully sterile (fertile in 0/5) (Figure 7D). At the recovery time of 4 months, 30.8% (4/13) of the male Sox30^loxp/loxp^ mice were fertile, while 100% (8/8) of both male Sox30^+/+^ and Sox30^−/+^ mice injected with Tam were fertile; the male Sox30**^−/−^** mice injected with solvent remained fully sterile (fertile in 0/8) (Figure 7D). At the recovery time of 8 months, 45% (9/20) of the male Sox30^loxp/loxp^ mice were fertile, 100% (10/10) of the male Sox30^+/+^ mice injected with Tam were fertile, 90% (9/10) of the male Sox30^−/+^ mice injected with Tam were fertile, and the male Sox30**^−/−^** mice injected with solvent remained fully sterile (fertile in 0/10) (Figure 7D). We then evaluated Sox30 expression in testes from the inducible system, and observed that the Sox30 expression was indeed restored in testes of Sox30^loxp/loxp^ mice (Figure 7E; Figure S8B). These data revealed that Sox30 re‐expression in adult Sox30^−/−^ mice can successfully reverse the pathological damage in testes, and restore germ‐cell meiosis, differentiation and Leydig cell proliferation.

To analyse whether the functional recovery of testis phenotypes and germ‐cell differentiation is associated with regulation of Cyp26b1, Stra8, Rec8 and Ctnnb1 in Sox30^loxp/loxp^ mice, we evaluated the expression of these proteins in testes from the inducible system. Rec8 and Stra8 were reactivated, and Cyp26b1 and Ctnnb1 were reinhibited on restoring *Sox30* expression (Figure 7E; Figure S8B). These results suggested that Sox30 is required for germ‐cell meiosis and differentiation by regulation of *Cyp26b1*, *Stra8*, *Rec8* and *Ctnnb1*.

## 3 DISCUSSION

Meiosis is a programme with two cell divisions but only one round of DNA replication to generate haploid gametes. In mammals, the timing and regulation of meiosis differ between the two sexes (Baltus et al., 2006; Feng et al., 2014; Handel & Schimenti, 2010; Spiller & Bowles, 2015). In the mouse embryonic ovary, the switch from mitosis to the meiosis of germ cells occurs at approximately 13.5 dpc. In mouse testis, the meiosis is initiated at approximately 8 dpp. Despite the recent identification of some meiosis inducing factors such as RA and *Msx1*/*2* (Bowles et al., 2006; Koubova et al., 2006; Le Bouffant et al., 2011; Raverdeau et al., 2012), it remains largely unclear how meiosis is specifically promoted and maintained in postnatal testes (Feng et al., 2014; Handel & Schimenti, 2010; Rossitto et al., 2015). In the present study, we identified *Sox30* as a male‐specific intrinsic factor that promotes or maintains germ‐cell meiosis, which partially explains the long‐standing issue of how meiosis is specifically promoted or maintained in postnatal testes.

In Sox30**^−/−^** mice, no spermatozoa and no elongated spermatids were found in testes at different developmental stages, whereas round spermatids were observed in testes at and before 6 months. These phenotypes appeared consistent with the results of previous studies (Bai et al., 2018; Feng et al., 2017; Zhang et al., 2018). However, in the present study, we observed a considerably different issue, in which round spermatids in Sox30**^−/−^** testes continuously decreased as mice aged from 1.5 months to 6 months post‐partum and completely disappeared at and after 8 months post‐partum. This different result indicates that Sox30 acts primary roles in the prophase of germ‐cell differentiation, which probably occurs at least before round spermatid formation. Furthermore, we revealed that *Sox30* deletion in mice results in spermatogenic cell arrest at the meiotic prophase; this arrested phase is the zygotene stage of spermatocytes. In zygotene, homologous chromosomes initiate the assembly of the synaptonemal complex (Lee & Hirano, 2011). Thus, it is reasonable to speculate that *Sox30* might disrupt the synaptonemal complex during meiosis of male germ cells. However, the exact role of *Sox30* in the synaptonemal complex remains unknown, and further studies are required to explore this issue.

Recent investigations have revealed that *Sox30* is essential for spermatogenesis (Bai et al., 2018; Feng et al., 2017; Zhang et al., 2018). However, the position of Sox30 expression in testes and the arrest period of germ‐cell development in Sox30‐deficient mice are controversial. Zhang et al. have shown that Sox30 is predominantly expressed in spermatids (Zhang et al., 2018), whereas Bai et al. have observed that Sox30 expression is restricted to meiotic spermatocytes and postmeiotic haploids (Bai et al., 2018). Feng et al. and Zhang et al. have revealed that germ cells are arrested at the postmeiotic round spermatids in *Sox30* knockout mice (Feng et al., 2017; Zhang et al., 2018), whereas Bai et al. have indicated the accumulation of meiotic diplotene spermatocytes, suggesting an impaired transition from meiosis to postmeiosis in *Sox30* knockout mice (Bai et al., 2018). In our present study, we observed that Sox30 expression is predominantly restricted to meiotic spermatocytes (high level) and round spermatids (relatively low level), especially to meiotic spermatocytes, using two Sox30 antibodies, which is additionally confirmed by another recent study (Roumaud et al., 2018). Particularly, we revealed that *Sox30* is a key factor for promoting or maintaining male germ‐cell meiosis, and deletion of *Sox30* resulted in impaired zygotene‐to‐pachytene transition. Interestingly, deletion of *Sox30* does not cause an immediate arrest of meiosis completely but becomes more apparent and severe in ageing mice. The specific reasons for this phenomenon need to be further investigated.

In the present study, the conclusion of the meiotic arrest of germ cells is distinct from previously reported in Sox30**^−/−^** mice (Bai et al., 2018; Feng et al., 2017; Zhang et al., 2018), but the arrest at zygotene of male germ cells in Sox30**^−/−^** mice is highly credible: (1) the number of round spermatids was not increased as mice aged in Sox30‐null testes, and instead, it was decreased and even completely disappeared in ageing mice. If the germ‐cell arrest occurs at round spermatids in Sox30**^−/−^** testes, the round spermatids in Sox30**^−/−^** testes should be accumulated at least not decreased or even disappeared; (2) DNA content analyses showed few cells harbouring one copy of the chromosome, and round spermatids also had one copy of chromosome; (3) there are some multi‐nucleated spermatogenic cells (XC cells) in testes of Sox30**^−/−^** mice, and if germ cells arrest at round spermatids in Sox30**^−/−^** testes, where did these multi‐nucleated cells appear from? (4) the primary spermatocytes were significantly accumulated in testes of Sox30**^−/−^** mice, as determined by the chromosome spread assay and primary spermatocyte marker analyses; (5) Sox30 expression is restricted to meiotic spermatocytes and round spermatids, especially to meiotic spermatocytes. These data strongly indicate that male germ cells are arrested at meiosis I in testes of Sox30^−/−^ mice, which is further highlighted via the mechanism of Sox30 function by regulating *Cyp26b1*, *Stra8* and *Rec8*.

At the molecular level, Sox30 promotes testicular germ‐cell meiosis by direct transcriptional activation of *Stra8* and *Rec8* expression and enhancing the RA‐signalling pathway *via* direct transcriptional inhibition of *Cyp26b1* expression. This activation of *Stra8* and *Rec8* by direct transcriptional regulation or via a general activation of RA‐signalling dependent transcriptional activity through direct transcriptional inhibition of *Cyp26b1* may provide a “fail‐safe” mechanism for Sox30 to facilitate meiosis, as well as ensure the strict execution of spermatogonial cell differentiation. Moreover, different domains of Sox30 are required for regulating activities of Cyp26b1, Rec8 and Stra8 promoters, which further strengthened the “fail‐safe” mechanism of Sox30 function by binding to different targets with different regions. These data further highlight the key role of Sox30 in male germ‐cell meiosis.

Our previous results have indicated that silencing SOX30 is possibly a crucial contributor to azoospermia disease (Han et al., 2020). However, the precise pathogenesis and mechanism of azoospermia induced by SOX30 silencing remain unclear. Based on the findings of the current investigation, the pathogenesis of azoospermia caused by SOX30 silencing could be that SOX30‐loss causes the abnormal arrest of germ‐cell meiosis at zygotene, leading to complete absence of spermatozoa and male infertility by directly regulating critical regulators of meiosis (*Cyp26b1*, *Stra8* and *Rec8*) and sex differentiation (*Sox9*, *Foxl2*, *Wnt4*, *Ctnnb1* and *Rspo1*). Thus, our data uncovered the pathogenesis of human azoospermia and advanced the current understanding of this disorder.

In the present study, the most interesting data generated involved experiments reversing the phenotype using an inducible knockout model. The restoration of germ‐cell differentiation and reappearance of spermatozoa were observed following inducible Sox30 expression in adult Sox30^−/−^ mice. However, it appears that despite analysis at 8 months post‐Tam (several complete cycles of spermatogenesis), a marked difference in testis phenotype persists between Sox30^+/+^ and Sox30^loxp/loxp^ mice. This could be mainly attributed to the incomplete restoration of Sox30 in Sox30^loxp/loxp^ mice owing to the efficiency of recombination. Further expression analyses revealed that the Sox30 expression was incompletely restored in testes of Sox30^loxp/loxp^ mice. These data suggest that testis phenotypes could be further ameliorated if the efficiency of recombination is improved in the restoration experiments.

In addition, our study revealed that the testis weight was significantly lower in Sox30**^−/−^** mice than in Sox30**^+/+^** mice, starting from 14 dpp, and Sox30 can regulate several critical genes of sex determination and differentiation, including the downregulation of *Sox9* and Sry, and upregulation of *Foxl2*, *Wnt4*, *Ctnnb1* and *Rspo1* from the RNA sequencing data in testes of Sox30**^+/−^** mice. Further experiments revealed that Sox30 significantly enhanced the activity of *Sox9* promoter and attenuated the activities of *Wnt4*, *Rspo1* and *Ctnnb1* promoters. Moreover, Sox30 suppressed Ctnnb1 expression by direct binding to its promoter, and Ctnnb1 was reinhibited on restoring *Sox30* expression in Sox30^−/−^ mice. These data indicate that Sox30 is strongly associated with sex differentiation even sex determination in mice. Additional investigations are required to clarify the sex ratio of the mutant mice, and the precise role and mechanism of Sox30 on sex differentiation even sex determination at early developmental stages.

In summary, our study revealed that *Sox30* plays an age‐related key role in promoting or maintaining the meiosis of germ cells in testes, and in sex differentiation by directly regulating *Stra8*, *Rec8*, *Cyp26b1* and *Ctnnb1*, and re‐expression of *Sox30* in adult Sox30^−/−^ mice can successfully restore germ‐cell meiosis and differentiation, and testis development (Figure 7F). This study advances our understanding of the regulation of male germ‐cell meiosis and differentiation, and the pathogenesis of azoospermia.

## 4 EXPERIMENTAL PROCEDURES

### 4.1 Cell lines

The cell lines were obtained from the Cell Bank of the Chinese Academy of Science (CBCAS, Shanghai, China). Details are provided in Supplemental experimental procedures.

### 4.2 Gene‐null mice

Sox30‐null mice were generated by the Model Animal Research Center of Nanjing University. All experiments were performed with permission from the Institutional Animal Care and Use Committee of Nanjing University and Army Medical University. Details are provided in Supplemental experimental procedures.

### 4.3 Fertility assays

Breeding assays were carried out, and details are provided in Supplemental experimental procedures.

### 4.4 Epididymal sperm count, RNA and protein extraction, and RT‐qPCR

Detailed methods are provided in Supplemental experimental procedures.

### 4.5 Restoration of sox30 repression in sox30^−/−^ mice by tamoxifen injection

Detailed methods are provided in Supplemental experimental procedures.

### 4.6 Histology haematoxylin and eosin staining, and transmission electron and optical microscopy

Detailed methods are provided in Supplemental experimental procedures.

### 4.7 Chromosome spread and immunostaining

Chromosome spread assays were performed as previously described (Peters et al., 1997). Detailed methods are provided in Supplemental experimental procedures.

### 4.8 Flow cytometry

Testicular cell suspensions were prepared as previously described (Rodríguez‐Casuriaga et al., 2009, 2014). Detailed methods are provided in Supplemental experimental procedures.

### 4.9 Terminal deoxynucleotidyl transferase‐mediated dutp nick end labelling assay

Testicular paraffin sections were prepared and the apoptotic cells were detected using in situ cell death detection kit, POD (Roche, Penzberg, Germany). Details are provided in Supplemental experimental procedures.

### 4.10 Spermatogenic cell proliferation assay

Spermatogenic cell proliferation was measured by performing the 5‐ethynyl‐2’‐deoxyuridine (EdU) assay. Detailed methods are provided in Supplemental experimental procedures.

### 4.11 RNA extraction, library construction and transcriptome sequencing

Detailed methods are provided in Supplemental experimental procedures.

### 4.12 Heatmap analysis

Detailed methods are provided in Supplemental experimental procedures.

### 4.13 Western blotting

Western blotting (WB) analysis was performed as previously described (Han et al., 2015). Detailed methods are provided in Supplemental experimental procedures.

### 4.14 Immunofluorescence

Detailed methods are provided in Supplemental experimental procedures.

### 4.15 Retinoic acid concentration

RA concentration in the testis was determined using mouse retinoic acid ELISA Kit (MyBioSource, San Diego, CA, USA MBS706971_48 T) according to the manufacturer's instructions. Details methods are provided in Supplemental experimental procedures.

### 4.16 Construction of vectors and cell transfection

Detailed methods are provided in Supplemental experimental procedures.

### 4.17 Site‐Directed mutagenesis assay

Sox30 HMG‐box constructs were mutated using a QuikChange Lightning Site‐Directed Mutagenesis Kit (Stratagene, La Jolla, CA, USA) as previously described (Han, Liu, et al., 2018). Detailed methods are provided in Supplemental experimental procedures.

### 4.18 Luciferase reporter assay

Detailed methods are provided in Supplemental experimental procedures.

### 4.19 Chromatin immunoprecipitation‐pcr assay

ChIP analyses were performed using a tissue ChIP Assay Kit (Epigentek, Bi County Blvd. Ste., Farmingdale, NY, USA, P‐2003) or a cell ChIP Assay Kit (Pierce, Rockford, lL, USA, 26156). Detailed methods are provided in Supplemental experimental procedures.

### 4.20 Statistical analysis

Statistical analyses were performed using SPSS 15.0 software (SPSS, Inc., Chicago, IL, USA). Detailed methods are provided in Supplemental experimental procedures.

## CONFLICT OF INTEREST

The authors declare that they have no potential conflicts of interest.

## AUTHOR CONTRIBUTIONS

F.H., J.L. and J.C. designed the project; F.H., L.Y., X.J., X.Z., N.Z., J.Y., X.H., W.L., Y.H. and H.C. performed the experiments; F.H., L.Y., X.J., Z.L. and Q.G. analysed the experimental data; F.H., Y.H. and F.G. performed the bioinformatic analysis; F.H., J.L., J.C. and W.O. wrote and revised the manuscript with comments from all authors.

## Supporting information

Fig S1‐8

Table S1‐2

Supplementary Material

## Data Availability

All the data are available in the main text and supplemental materials.
