# Corrigendum to: Identification of SRY‐box 30 as an age‐related essential gatekeeper for male germ‐cell meiosis and differentiation

**DOI:** 10.1111/acel.13728

**Published:** 2022-10-07

Fei Han, Li Yin, Xiao Jiang, Xi Zhang, Ning Zhang, Jun‐tang Yang, Wei‐ming Ouyang, Xiang‐lin Hao, Wen‐bin Liu, Yong‐sheng Huang, Hong‐qiang Chen, Fei Gao, Zhong‐tai Li, Qiao‐nan Guo, Jia Cao, Jin‐yi Liu, *Aging Cell*, 2021;20:e13343. https://doi.org/10.1111/acel.13343

In the published version of Han, F., et al. (2021), the authors noticed that the two genes “*Foxl2*” and “*Rec8*” has been mislabelled with an opposite place in the histograms of quantitative PCR in Figure 5(b).

The corrected figure is shown below.

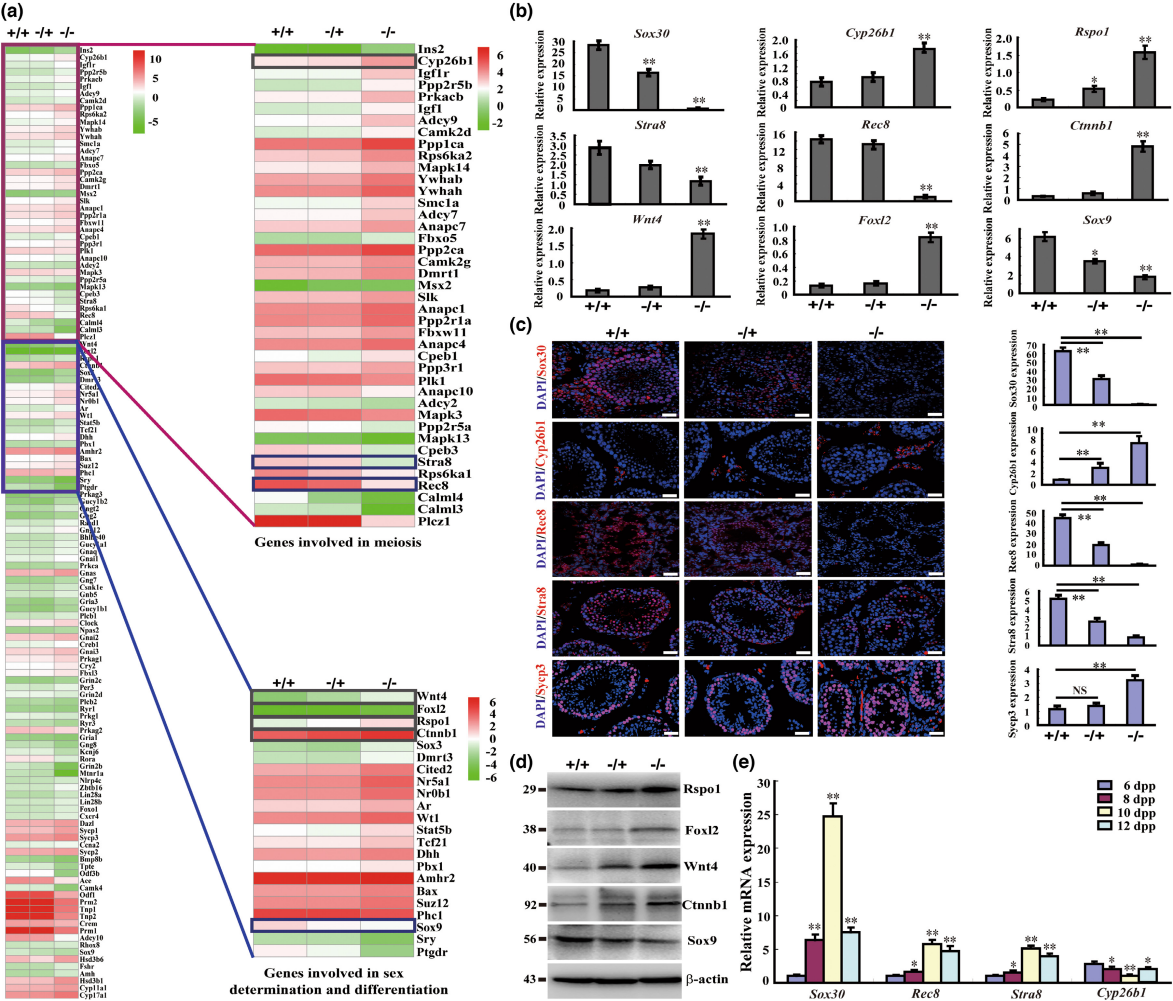

The authors apologize for this error.

