# Supplementary material for: Identification of SRY‐box 30 as an age‐related essential gatekeeper for male germ‐cell meiosis and differentiation
Source: Aging Cell. 2021 Mar 15;20(5):e13343. doi: 10.1111/acel.13343 (PMC8135013; doi:10.1111/acel.13343)
Supplement: Supplementary file 1 — Fig S1‐8 [file ACEL-20-e13343-s001.doc]

**Supplemental figures and figure legends**


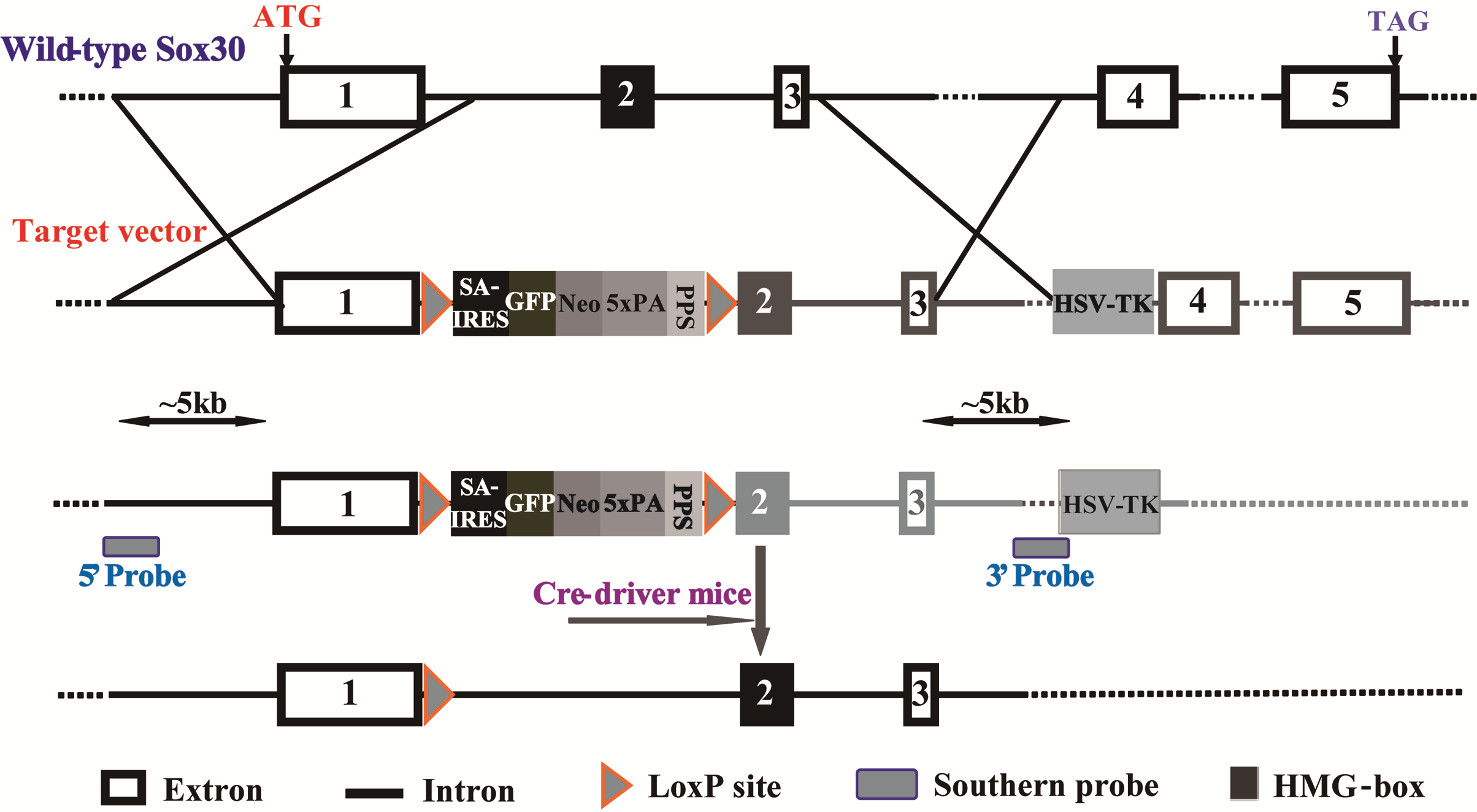


**Figure S1 Generation of *Sox30*-null mice by homologous recombination**

Targeted disruption of mouse *Sox30* was generated by knockin strategy. The LoxP-SA-IRES-GFP-NEO-STOP(poly-A)-PPS-LoxP cassette was introduced to *Sox30* between Exon1 and Exon2. The numbered boxes denote Sox30 exons.

**
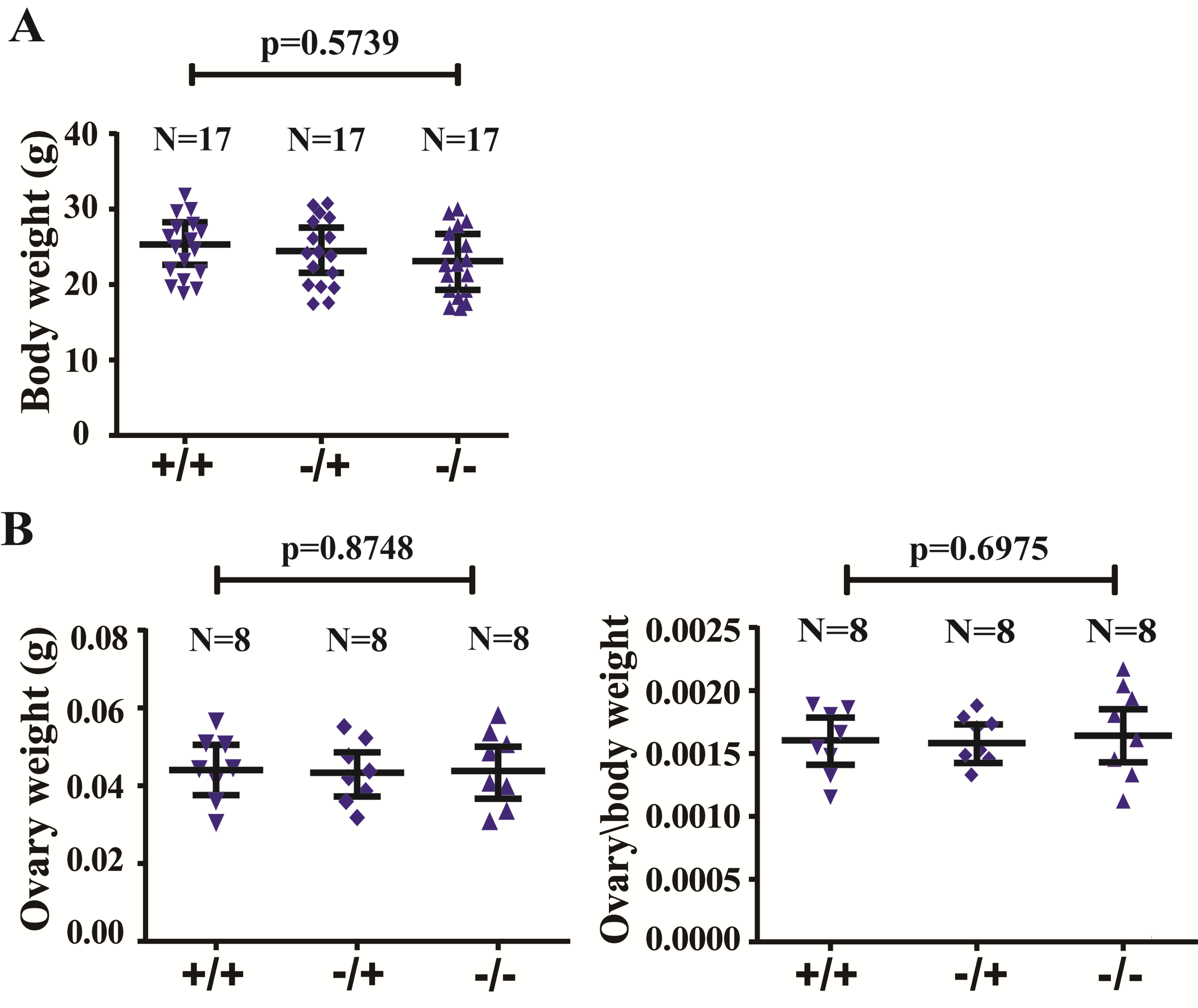
**

**Figure S2 The effect of Sox30 on body growth and ovarian development was determined in mice.**

(A) The body weight was analyzed in Sox30**^+/+^** (+/+), Sox30**^-/+^** (-/+) and Sox30**^-/-^** (-/-) adult (4 months) mice. (B) The ovary weight and ovary/body weight were evaluated in Sox30**^+/+^**, Sox30**^-/+^** and Sox30**^-/-^** adult (4 months) mice.

**
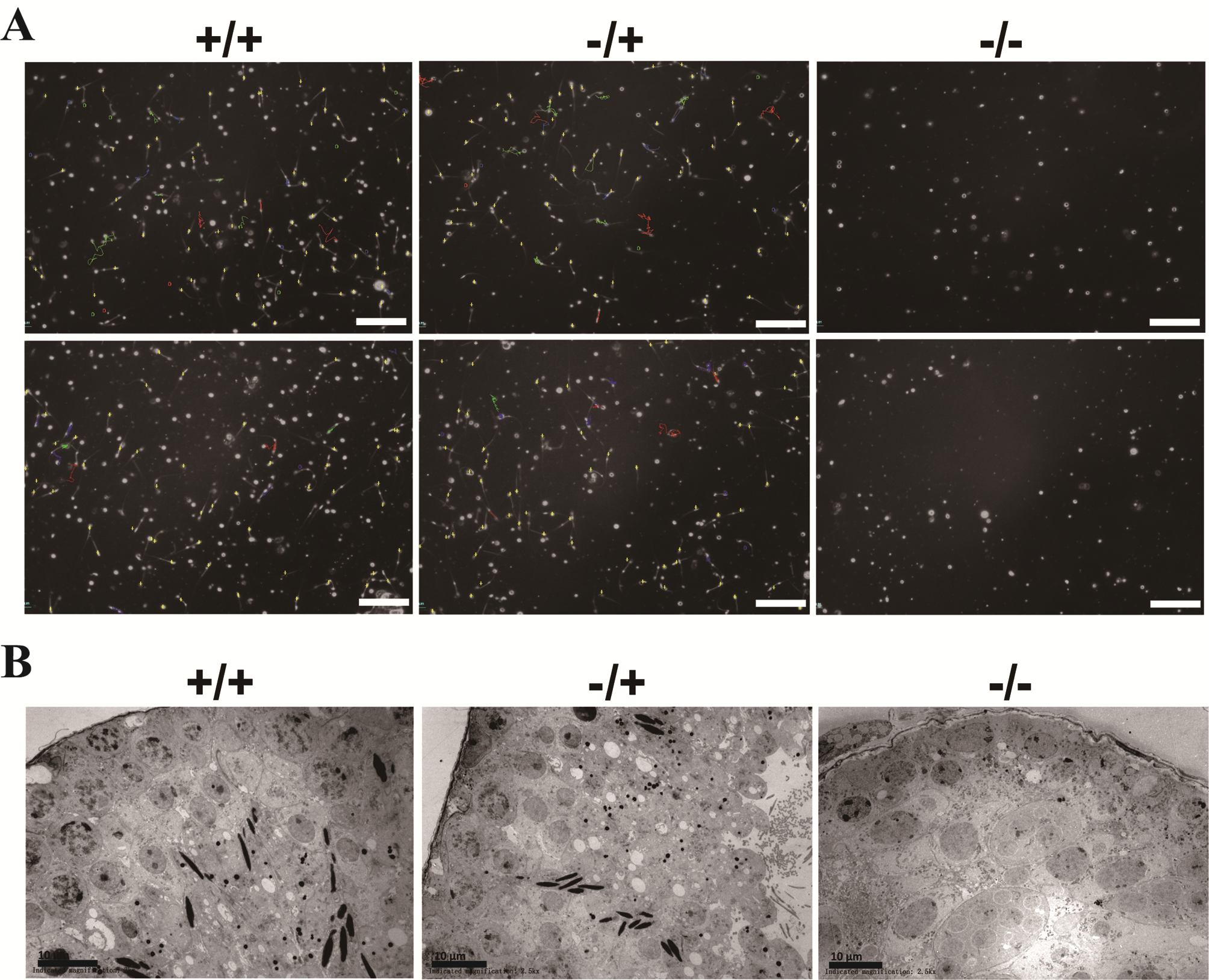
**

**Figure S3 Sox30 deletion caused scarce spermatids and complete absence of spermatozoa**

(A) Analyses of spermatozoa in the epididymides of Sox30**^+/+^**, Sox30**^-/+^** and Sox30**^-/-^** mice were performed by sperm class analyzer (SCA) system. Scale bars are 50µm. (B) Morphological examinations of the testes were performed in Sox30**^+/+^** , Sox30**^-/+^** and Sox30**^-/-^** mice by transmission electron microscopy (TEM). Scale bars are 10µm.

**
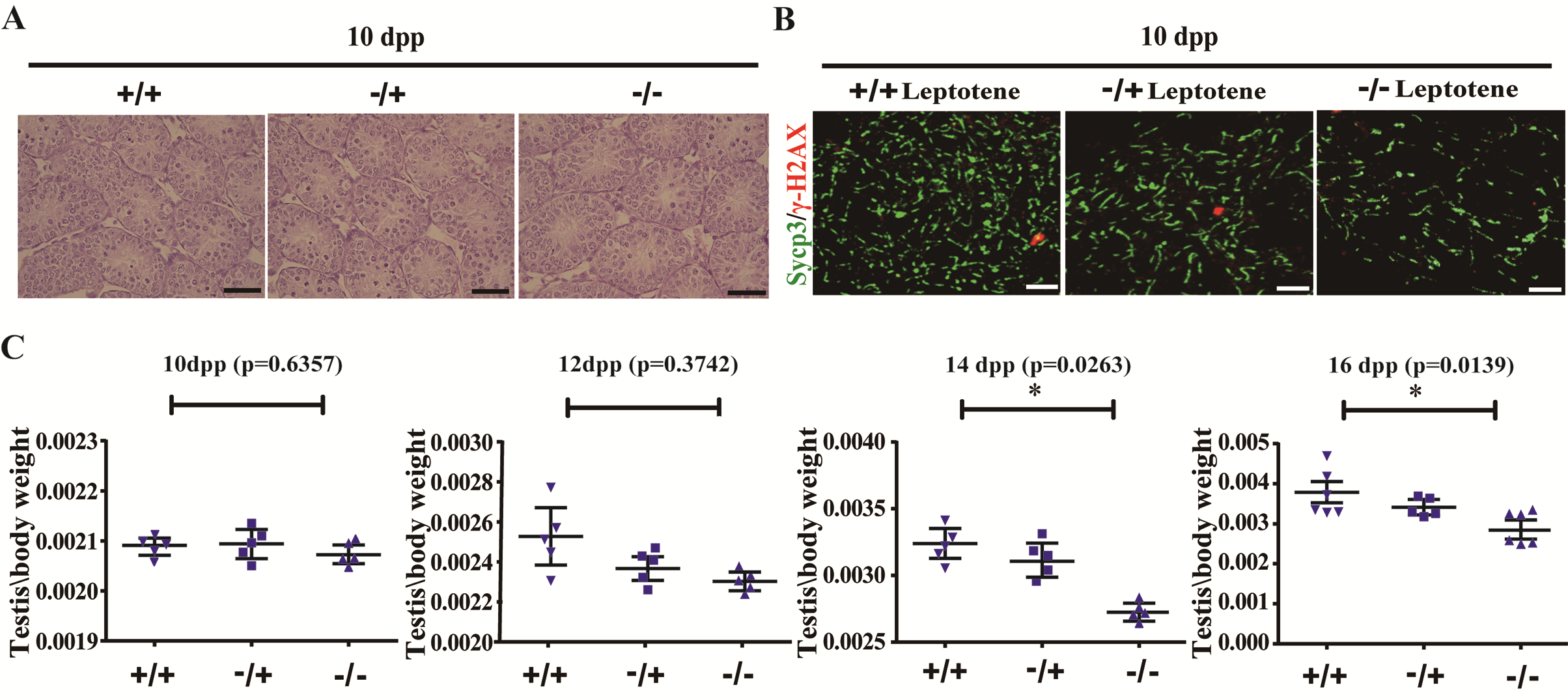
**

**Figure S4 H&E staining, chromosome spread and weight of the testes in mice with indicated genotypes**

(A) H&E staining of histological sections of the testes was shown in Sox30^+/+^, Sox30^-/+^ and Sox30^-/-^ mice at 10dpp stage. Scale bars are 50µm. (B) Chromosome spread staining of Sycp3 and γ-H2AX for spermatocytes was examined in the testes from Sox30^+/+^, Sox30^-/+^ and Sox30^-/-^ mice at 10dpp stage. Scale bars are 20µm. (C) The testis/body weight was analyzed in Sox30^+/+^, Sox30^+/-^ and Sox30^-/-^ mice at different development stages. The 10dpp, 12dpp, 14dpp and 16dpp represent the mice at 10, 12, 14 and 16 days post-partum, respectively. The “*” represent p value less than 0.05.

**
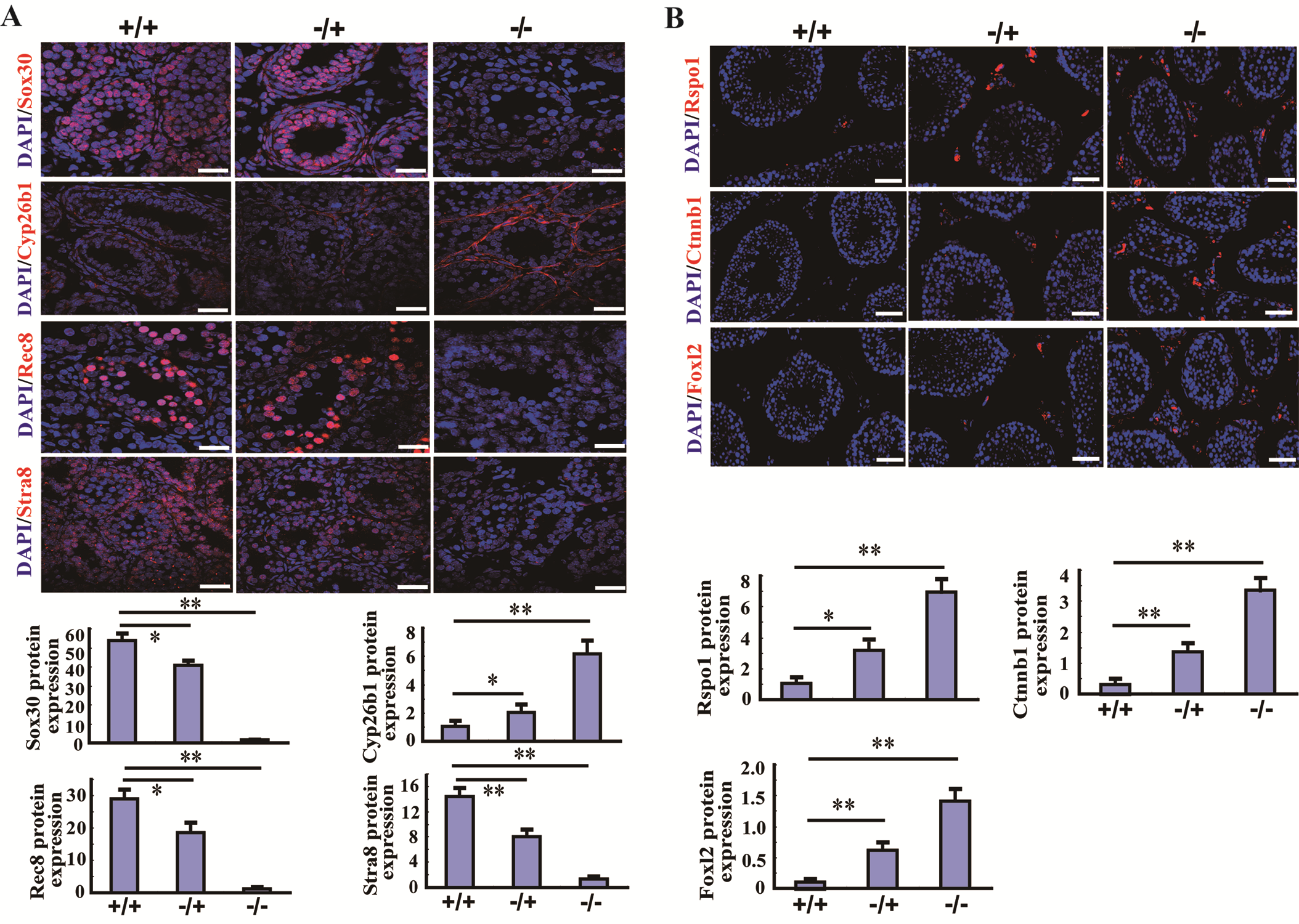
**

**Figure S5 Sox30 is associated with** **critical regulators of meiosis and sex differentiation in testes**

(A) The Sox30, Cyp26b1, Rec8, Stra8 and Sycp3 expression was tested by immunofluorescence staining in testes of Sox30**^+/+^**, Sox30^-/+^ and Sox30**^-/-^** mice at 12 dpp. The “**” represent p value less than 0.01. The “*” represent p value less than 0.05. Scale bars are 20µm. (B) The Rspo1, Ctnnb1 and Foxl2 expression was tested by immunofluorescence staining in testes of Sox30**^+/+^**, Sox30^-/+^ and Sox30**^-/-^** mice at adult (3 months). The “**” represent p value less than 0.01. The “*” represent p value less than 0.05. Scale bars are 20µm.

**
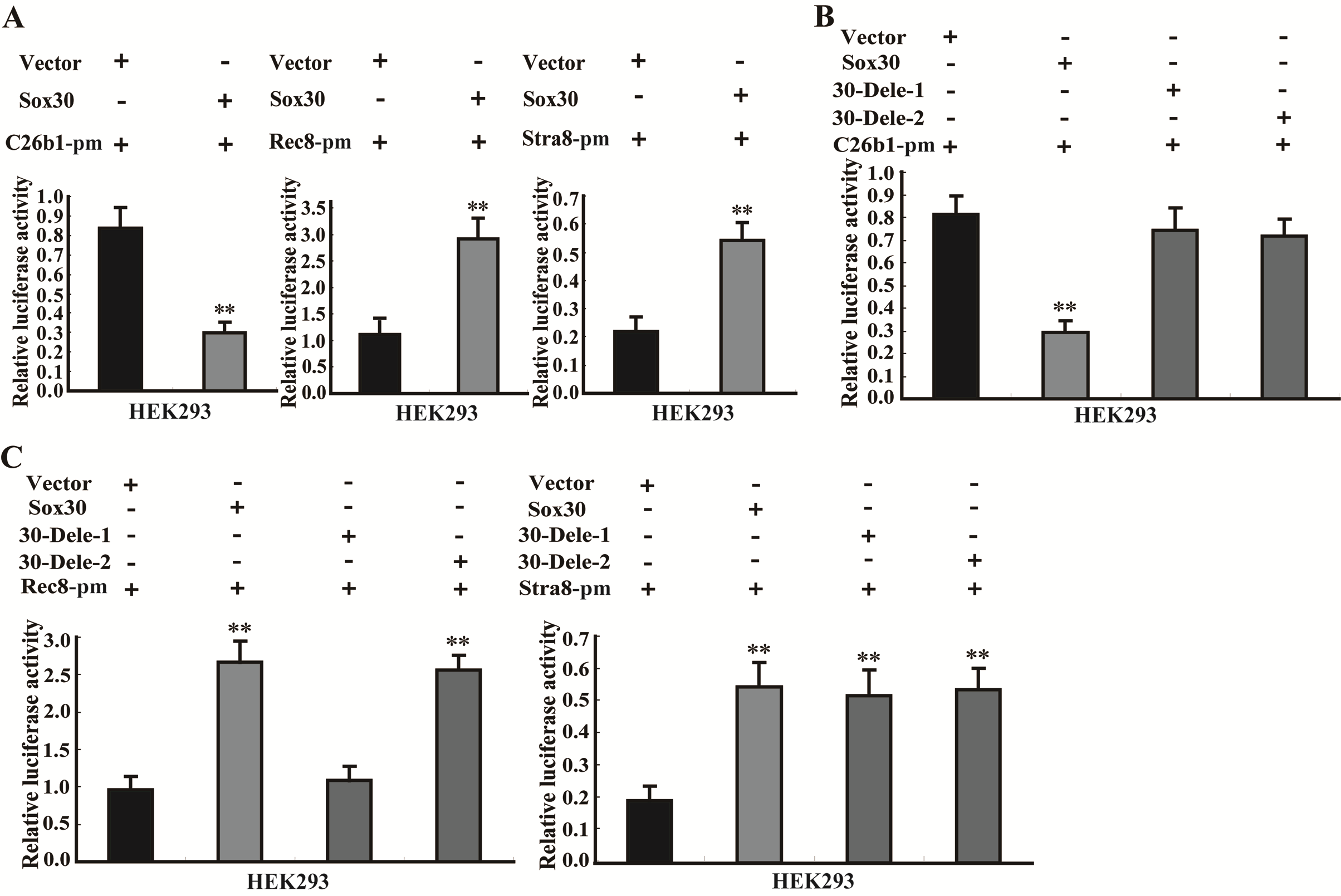
**

**Figure S6 Sox30 regulates the expression of Cyp26b1, Rec8 and Stra8 gene**

(A) Luciferase reporter assays were used to analyze Sox30-induced inhibition of Cyp26b1 promoter and activation of Rec8 and Stra8 promoters in HEK293 cells. Results were normalized with internal controls and presented as averages with SEM from three experiments. The “**” represent p value less than 0.01. (B, C) The effect of Sox30 with or without HMG-box and/or C-terminal domains on Cyp26b1, Rec8 and Stra8 promoter activity was determined in HEK293 cells. The “**” represent p value less than 0.01.

**
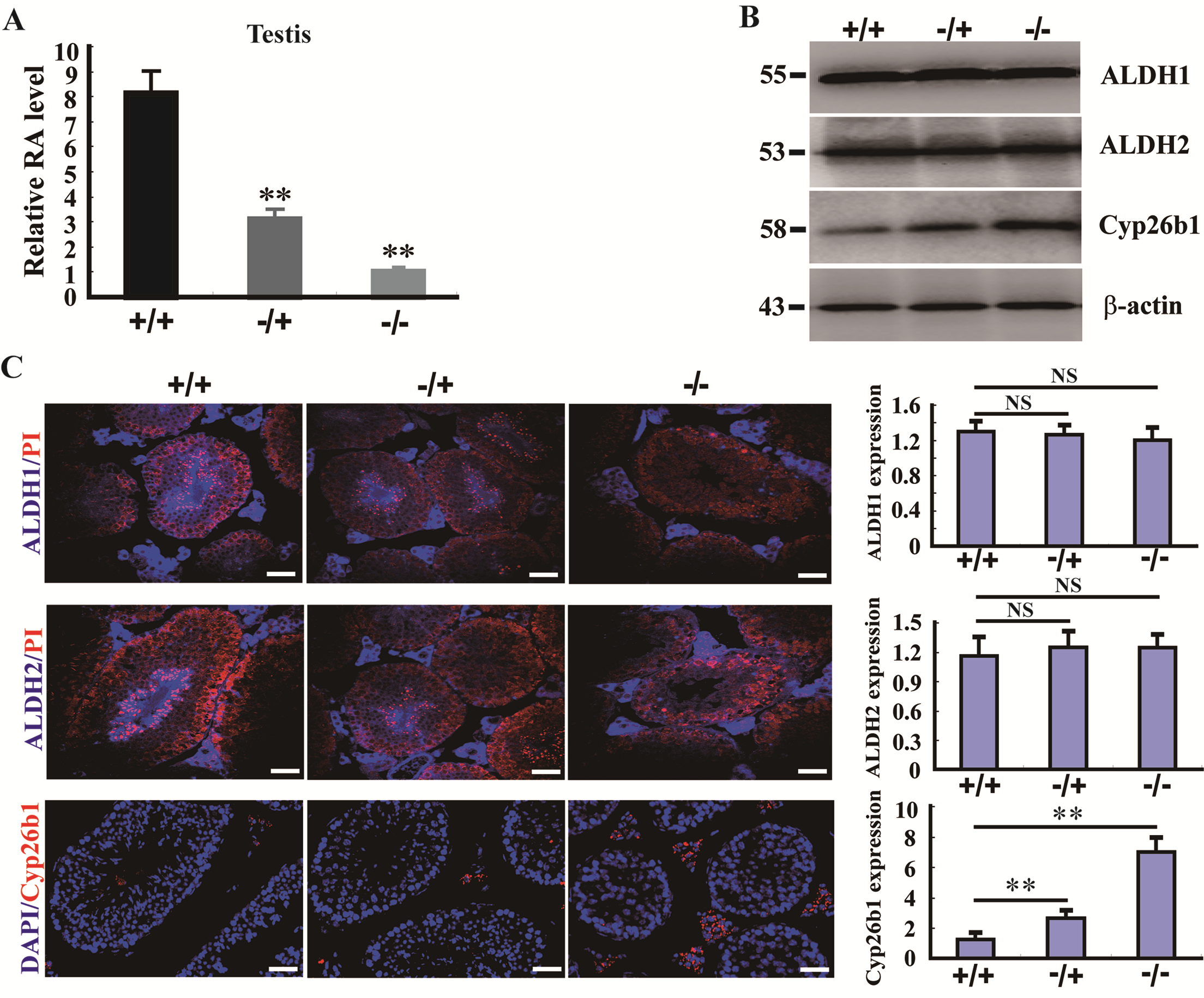
**

**Figure S7 RA levels were reduced in Sox30-null testes because of increased degradation**

(A) RA levels were markedly decreased in adult (3 months) testes of Sox30**^+/-^** and Sox30**^-/-^** mice compare with in the testes of Sox30**^+/+^** mice. The “**” represent p value less than 0.01. (B) The two major enzymes ALDH1 and ALDH2 responsible for RA production and Cyp26b1 were determined by WB in adult (3 months) testes of Sox30**^+/+^**, Sox30**^+/-^** and Sox30**^-/-^** mice. The β-actin was used as an internal control. (C) The two major enzymes ALDH1 and ALDH2 responsible for RA production and Cyp26b1 were examined by immunofluorescence staining in adult (3 months) testes of Sox30**^+/+^**, Sox30^-/+^ and Sox30**^-/-^** mice. The “**” represent p value less than 0.01. The “NS” represent p value more than 0.05. Scale bars are 20 µm.

**
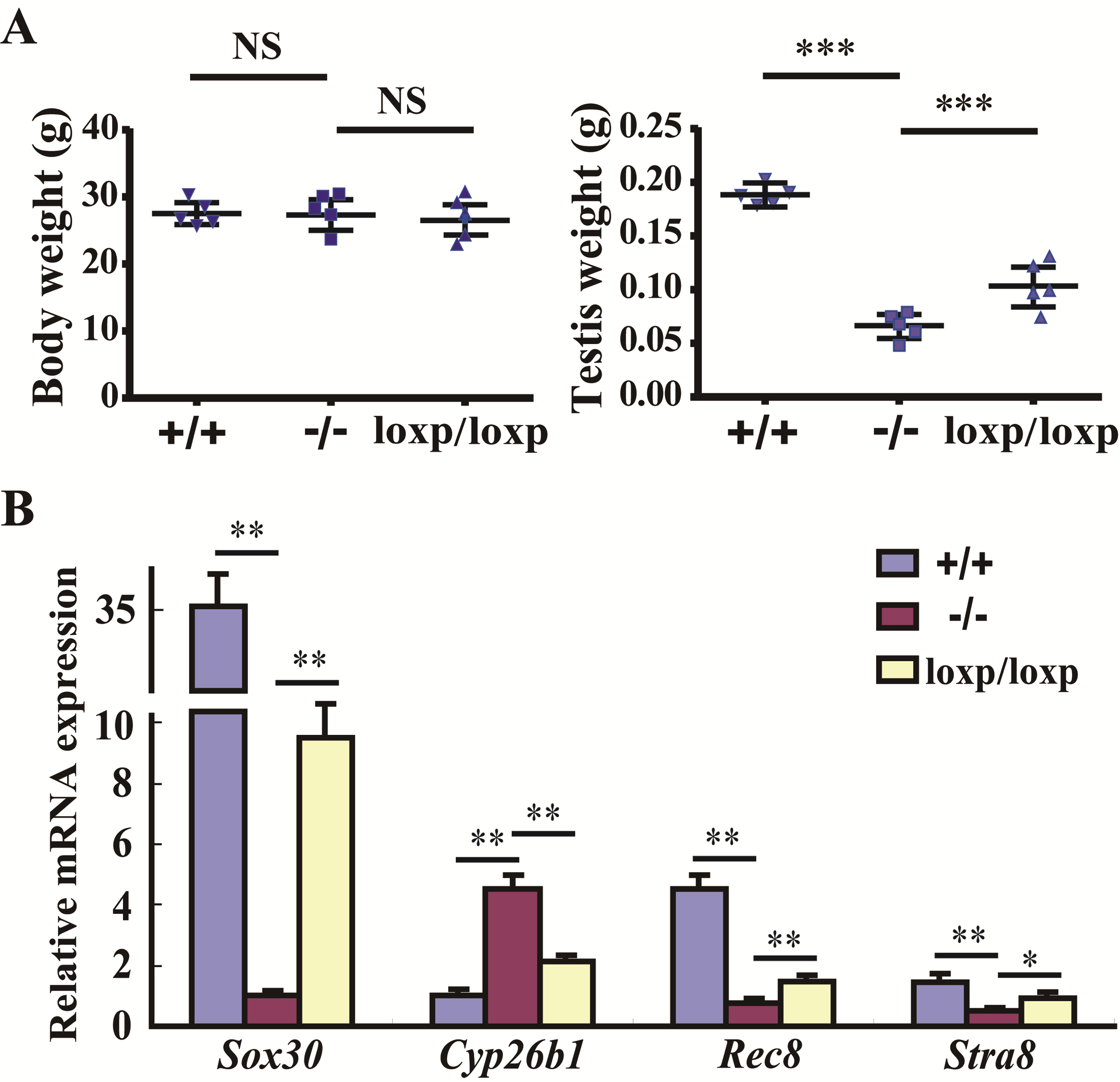
**

**Figure S8 Sox30 is required for male germ cell meiosis by regulation of Cyp26b1, Stra8 and Rec8**

(A) The weight of body and testis was analyzed in Sox30**^+/+^** (n=5), Sox30**^-/-^** (n=5) and Sox30^loxp/loxp^ (n=5) mice. +/+, Sox30^+/+^ mice injected with tam; -/-, Sox30**^-/-^** mice injected with solvent; loxp/loxp, Sox30^-/-^ mice injected with tam. The “***” represent p value less than 0.001. The “NS (no significance)” represent p value more than 0.05. (B) The expression levels of Sox30, Cyp26b1, Stra8 and Rec8 were analyzed by RT-qPCR in the testes of Sox30**^+/+^**, Sox30**^-/-^** and Sox30^loxp/loxp^ mice. The β-actin was used as an internal control. The “**” represent p value less than 0.01. The “*” represent p value less than 0.05.
