# Supplementary material for: Identification of SRY‐box 30 as an age‐related essential gatekeeper for male germ‐cell meiosis and differentiation
Source: Aging Cell. 2021 Mar 15;20(5):e13343. doi: 10.1111/acel.13343 (PMC8135013; doi:10.1111/acel.13343)
Supplement: Supplementary file 2 — Table S1‐2 [file ACEL-20-e13343-s002.doc]

**Supplemental tables**

***Table S1 Putative binding sites of Sox30 were predicted in promoters of Cyp26b1, Stra8, Rec8, Sox9, Foxl2, Wnt4, Ctnnb1 and*** Rspo1

| **Genes** | **Score** | **Relative score** | **Start** | **End** | **Predicted site sequence** |
| --- | --- | --- | --- | --- | --- |
| **Cyp26b1-pm**  (-1891~ +144bp) | 8.432 | 0.81169 | 1104 | 1119 | CTGGCATAATTGAATT |
|  | 8.778 | 0.81936 | 1334 | 1349 | GAAGGACAATCAGGAT |
|  | 9.111 | 0.82673 | 1625 | 1640 | CATGCACAATGGTCTG |
| **Stra8-pm**  (-1900~+100bp) | 8.447 | 0.81203 | 786 | 801 | AAAAAAAAATGAAGTT |
|  | 7.959 | 0.80122 | 1544 | 1559 | AGAGAAAAATGAAGAT |
| **Rec8-pm**  (-1800~ +136bp) | 8.147 | 0.80538 | 137 | 152 | AAAAAAGAATGAAAGA |
|  | 8.227 | 0.80715 | 320 | 335 | ACAAAACAAAAAAATA |
|  | 9.592 | 0.83739 | 702 | 717 | GAGAGATAATGAAATC |
| **Sox9-pm**  (-2000~ +400bp) | 10.765 | 0.86337 | 262 | 641 | AATGAAATTGTTTATC |
|  | 8.904 | 0.82214 | 1511 | 1526 | ATTTCCATTGCTGTAA |
|  | 8.847 | 0.82088 | 276 | 291 | ATGGCTATTGTTTGTA |
|  | 8.189 | 0.80631 | 1284 | 1299 | GTTTTCATTGATTCCC |
|  | 8.050 | 0.80322 | 416 | 431 | AAATAACAAATGCCCA |
| **Foxl2-pm**  (-2000~ +150bp) | 9.492 | 0.83517 | 641 | 656 | GAAGGACAAAGGAAGA |
|  | 9.379 | 0.83265 | 322 | 337 | CAGACCATTATCCTTC |
| **Ctnnb1-pm**  (-2000~ +200bp) | 11.564 | 0.88106 | 827 | 842 | ATGTGACAATTAAAAT |
|  | 10.906 | 0.86649 | 1332 | 1347 | GGACAACAATGGGGCC |
|  | 9.876 | 0.84368 | 1167 | 1182 | GGACCTATTGTTTACT |
|  | 9.086 | 0.82617 | 182 | 197 | GGTTTCTTTATTCATC |
|  | 8.631 | 0.81610 | 95 | 110 | GGGTCAATTATTTTAG |
|  | 8.559 | 0.81450 | 1234 | 1249 | GTTGCCATTGTTTGGG |
|  | 10.826 | 0.81140 | 115 | 130 | TAATTTTATAAAACCA |

The putative sites were predicted with setting 80% in *Cyp26b1*, *Stra8*, *Rec8*, *Sox9*, *Foxl2* and *Ctnnb1* promoters (no sites were found in *Rspo1* and *Wnt4* promoters) using Jaspar at <http://jaspar.genereg.net/>.

**Table S2 Primer sequences were used in this study**

| **Genes** | **Primer sequence (5’-3’)** | **Primer purpose** |
| --- | --- | --- |
| Sox30-F | CCCATTCCACACTCACACGTCTA | RT-qPCR analysis |
| Sox30-R | AACCAAGACATTCTGGCATTGAACT |
| Stra8-F | CTGTTGCCGGACCTCATGG |
| Stra8-R | TCACTTCATGTGCAGAGATGATG |
| Cyp26b1-F | ACATGCTGTTTGAAGGCTTGG |
| Cyp26b1-R | TCTCTCCGATGAGCGGGAAT |
| Rec8-F | CCGTTTGGTGAAGCGTGAAT |
| Rec8-R | AAGCTGGGCGGAGAGATAGA |
| Sox9-F | GGCTCCTACTACAGTCACGC |
| Sox9-R | AGACTGGTTGTTCCCAGTGC |
| Foxl2-F | AACACCGGAGAAACCAGACC |
| Foxl2-R | CGTAGAACGGGAACTTGGCT |
| Wnt4-F | AGGAGACGTGCGAGAAACTC |
| Wnt4-R | TCCGGAACTGGTATTGGCAC |
| Rspo1-F | GAGACAGAGGCGGATCAGTG |
| Rspo1-R | CGGATGTCGTTCCTCTCCAG |
| Ctnnb1-F | ACTTGCCACACGTGCAATTC |
| Ctnnb1-R | ATGGTGCGTACAATGGCAGA |
| Tnp1-F | ACAAGGGCGTCAAGAGAGGT |
| Tnp1-R | CATCACAAGTGGGATCGGTA |
| Tnp2-F | TCACACCAGTAACCAGTGCAA |
| Tnp2-R | CAGGTGAGTGTCGAGAGTGC |
| Prm2-F | GAAGGCGGAGGAGACACTC |
| Prm2-R | GGGAGGCTTAGTGATGGTG |
| Actin-F | GGAGATTACTGCTCTGGCTCCTA | RT-qPCR analysis  (Internal control) |
| Actin-R | GACTCATCGTACTCCTGCTTGCTG |
| Gapdh-F | TGTGTCCGTCGTGGATCTGA |
| Gapdh-R | TTGCTGTTGAAGTCGCAGGAG |
| Cyp26b1-pF | CGGGGTACCTAGAAAGGCTTGGAGTCCTTCCAAGAC | Amplification of promoter |
| Cyp26b1-pR | CCCAAGCTTCCAAACACTTTGCCCAGGAGGAATTA |
| Rec8-pF | CGACGCGTGCAGAGGCAAGTAAATCGCTAAATTTGG |
| Rec8-pR | CCCAAGCTTGAGACACAGAACAGATCTTCAAAGAAGACTGA |
| Stra8-pF | CCCAAGCTTTGGAAGTCCCTACCATTCAGGAG |
| Stra8-pR | CGACGCGTCTTACCTTTAAGGCCACCACGG |
| Sox9-pF | CGACGCGTTGGATGTATTCATGAACAAATACTTAATAAA |
| Sox9-pR | GATCTCGAGATGAAGGGGTCCAGGAGATTCAT |
| Foxl2-pF | GGGGTACCTATCCTGGTGGTTAGGCCGGGTTTG |
| Foxl2-pR | GAAGATCTGACGCCTCGGCCTCTTTGACTGC |
| Wnt4-pF | GGGGTACCTGTGTCCCCTCCCTGGGTCTCACT |
| Wnt4-pR | GATCTCGAGAGAACACGGCGAAGACGAGGAGTC |
| Rspo1-pF | GGGGTACCCAGACACAGCCAACGGCAGGCA |
| Rspo1-pR | GATCTCGAGGACTCACCTGGCGCAGCGGC |
| Ctnnb1-pF | GGGGTACCACTTTATGTCGAACCCATTGGGCTGATCT |
| Ctnnb1-pR | GAAGATCTTCGGCCTCCTGCACTGACGGCT |
| Rec8p-F | TCCAGGGCTACACAGAGAAACCCT | ChIP-PCR analysis |
| Rec8p-R | CTCTTCAGAGTTGCACAGCAGCCT |
| Cyp26p-F | CATTGCTCCTAGGACTTCAGGACC |
| Cyp26p-R | GAATGGACGTTTAAGCAGGAGTGC |
| Stra8p-F | GGCAATGCCACCTATAACTTTCTCT |
| Stra8p-R | TCTCTGCTTTTTTAGTTGGGAATCC |
| Ctnnb1p-F | TGGAGACAGCCAATTCACCC |
| Ctnnb1p-R | TGTGTTGCTGAGAGGACCTG |
