# Supplementary material for: Identification of SRY‐box 30 as an age‐related essential gatekeeper for male germ‐cell meiosis and differentiation
Source: Aging Cell. 2021 Mar 15;20(5):e13343. doi: 10.1111/acel.13343 (PMC8135013; doi:10.1111/acel.13343)
Supplement: Supplementary file 3 — Supplementary Material [file ACEL-20-e13343-s003.doc]

**Experimental procedures**

**Cell lines**

The mouse NIH3T3 and human HEK293 cell lines were obtained from the Cell Bank of Chinese Academy of Science (CBCAS, Shanghai, China) cultured in DMEM (HyClone, Logan, UT, USA) media supplemented with 10% fetal bovine serum (FBS, Gibco, Gaithersburg, MD, USA) and incubated in 5% CO_2_ at 37°C. These cells lines have been authenticated and tested for mycoplasma contamination by CBCAS.

**Gene-null mice**

Sox30-null mice were generated by the Model Animal Research Center of Nanjing University. Briefly, targeted gene including homologous arms was retrieved from the BAC vector, and the LoxP-SA-IRES-GFP-NEO-STOP-PPS-LoxP cassette was introduced between Exon1 and Exon2 of Sox30 gene. The targeting vector was confirmed by PCR, enzyme digestion and sequencing, and was electroporated into C57BL/6 ES cells. The recombinants were selected by G418 and ganciclovir (Ganc). The positive clones were chosen for microinjection to generate chimeras, which were crossed with C57BL/6 mice to produce heterozygous. To restore Sox30 expression, we generated mice expressing Cre recombinase by breeding Sox30 heterozygous (Sox30**^+/-^**) mice with B6.Cg-Tg (CAG-cre/Esr1)5Amc/JNju tool mice (Stock No: 004682, The Jackson Laboratory) to produce Sox30-KI and Cre double positive heterozygote mice. Tamoxifen-inducible Cre-mediated recombination will result in deletion of the floxed sequences in the offspring of these mice. Mice were maintained in a specific pathogen-free unit under a 12h light/12h dark cycle with ad libitum access to water and food. All experiments were carried out with the permission of the Institutional Animal Care and Use Committee of Nanjing University and Army Medical University.

**Fertility assays**

Breeding assays with Sox30**^+/+^**, Sox30**^+/-^** and Sox30**^-/-^** mice or Sox30**^+/+^** male mice injected with tam, Sox30**^-/+^** male mice injected with tam, Sox30**^-/-^** male mice injected with tam (Sox30^loxp/loxp^) and the control Sox30**^-/-^** male mice injected with solvent were carried out. The mice (2 months old) of each sex for different genotypes were mated with two known fertility wild-type mice for a minimum of 2 months. Each male recovery mouse (two months old when injected with tam or solvent) at 2, 4 and 8 months after tam injection was mated with two wild-type females with known fertility for a minimum of 2 weeks. If progeny were born, the line was defined as fertile.

**Epididymal sperm count**

The epididymis was dissected from adult mice, and cut into pieces in 0.5 ml of medium incubating at 37°C for 10 min. Each sample (10µl) was transferred to a Glodcyto standard count chamber slide (Microptic S.L., Barcelona, Spain), warmed on microscope heating stage. Sperms on the slides were observed and counted at least in 5 random fields by SCA system (Microptic S.L., Barcelona, Spain).

**RNA and protein extraction**

Total RNA of cells or testis tissues was extracted using TRIzol Reagent (Invitrogen) and treated with DNase I to eliminate the genomic DNA contamination. Complementary DNA was synthesized using the GoScript^TM^ Reverse Transcription System (Promega) and stored at -20 °C. The protein was extracted using a lysis buffer (Beyotime, Shanghai, China) containing complete protease inhibitor (Roche, Mannheim, Germany), and stored at -80 °C after centrifugation.

**RT-qPCR**

RT-qPCR (quantitative reverse transcription polymerase chain reaction) was performed using GoTaq® qPCR Master Mix (Promega, USA) and CFX96™ Real-Time PCR Detection System (Bio-Rad Laboratories) as previously described (Han et al. 2015). The mRNA expression was calculated by 2^-ΔΔCT^. All assays were performed in triplicate for three times. The primers used in this study were listed in Supplemental Table S2.

**Restoration of Sox30 repression in Sox30^-/-^ mice by tamoxifen injection**

To induce Cre-mediated Sox30 expression, tamoxifen (tam, Sigma-Aldrich, St Louis, MO, USA) was injected into adult (2 months) mice once a day by intraperitoneal (i.p.) for 5 days with 1 mg/mice to remove the insertion cassette via activating the Cre recombinase (the control Sox30**^-/-^** mice were injected with solvent via i.p following the same steps). The mice were sacrificed at 8 months after the first injection, and the histology of testes was examined by microscopy.

**Histology haematoxylin and eosin stainin*g***

Testes were dissected and immediately fixed in Bouin’s fluid. The fixed testes were dehydrated, embedded in paraffin and cut into 5 µm thick sections. These sections were de-waxed, rehydrated and stained with haematoxylin and eosin (H&E).

**Transmission electron and optical microscopy**

Testes were dissected, cut into 1mm cubes and placed into fresh 2.5% (w/v) glutaraldehyde overnight at 4°C. The testis tissues were washed in phosphate buffer saline (PBS) post-fixed for 1h in 1% osmium tetroxide at room temperature, dehydrated through a graded series of ethanol and acetone, and infiltrated with and embedded in Epon 812. Sections (100 nm) were obtained with an ultramicro-tome, stained with uranyl acetate and lead citrate, and observed under a JEM-1400 Plus transmission electron microscope (JEOL, Japan).

**Chromosome spread and immunostaining**

Chromosome spread assays were performed as previous study (Peters et al. 1997). Briefly, testes were dissected from mice quickly and placed into PBS. Seminiferous tubules were cut into pieces after removing the tunica albuginea, and incubated in hypotonic extraction buffer (50 mM sucrose, 0.5 mM dithiothreitol, 5 mM EDTA, 17 mM trisodium citrate dehydrate, 30 mM Tris/HCl and 0.5 mM phenylmethylsulfonyl fluoride, pH 8.2,) for 1 hour (h), followed by centrifugation at x100g for 5 minutes at room temperature. The precipitate was suspended by pipetting thoroughly in 180 µL of 100 mM sucrose/HCl (pH 8.2) in which the debris was removed. An aliquot of 30 µL suspension was spread onto glass slides coated with freshly prepared fixative containing 1% paraformaldehyde/10 mM sodium borate (pH9.2) and 0.15% Triton X-100, fixed for 2 h in a moist chamber at room temperature, and dried completely. For immunostaining, the slides were washed in TBST, boiled in 10 mM citric acid/HCl (pH 6.0) for 15 minutes and washed again in TBST. Spread chromosomes were blocked for 1.5 h at room temperature in TBST with 0.5% BSA and 10% normal goat serum (Invitrogen), were incubated with primary antibodies overnight at 4°C, and washed in TBST. The spread chromosomes were probed for 2h with fluorescent secondary antibodies at room temperature, washed in TBST and observed under fluorescence microscope.

**Flow cytometry**

Testicular cell suspensions were prepared as previously described (Rodríguez-Casuriaga et al. 2009; Rodríguez-Casuriaga et al. 2014). Briefly, testes were dissected, placed into 35 mm dishes containing ice-cold separation medium (10% v/v fetal calf serum in Dulbecco’s Modified Eagle’s medium with high glucose and L-glutamine) and cut into pieces after removing the tunica albuginea. Cells were harvested, washed with PBS and resuspended with binding buffer. The cell suspension was stained with Vybrant™ DyeCycle™ Orange (Invitrogen-Life Technologies, Carlsbad, CA) at a final concentration of 10 μM for 1h at 37°C, and sequentially passed through 50 μm Filcon filter units (BD Biosciences, Franklin Lakes, NJ, USA). The samples were analyzed by FACSCalibur System (BD) and the cell cycle profiles were analyzed using the ModFit software (Verity Software House, Topsham, ME, USA). The assays were carried out in triplicate for three times.

**Terminal deoxynucleotidyl transferase-mediated dUTP nick end labeling assay**

Testicular paraffin sections were prepared and the apoptotic cells were detected using in situ cell death detection kit, POD (Roche, Penzberg, Germany). The terminal deoxynucleotidyl transferase-mediated dUTP nick end labeling (TUNEL) was performed according to the manufacturer’s instructions. Thereafter, the sections were followed by incubation in diaminobenzidine substrate (Roche) and the reaction was observed under a microscope. Finally, the nuclei were counterstained with hematoxylin buffer. The assays were carried out for three independent experiments.

**Spermatogenic cell proliferation assay**

The spermatogenic cell proliferation was measured by 5-ethynyl-2’-deoxyuridine (EdU). The mice were injected with 100 µg of EdU in PBS intraperitoneally and the spermatogenic cell proliferation assays were performed after incorporation 72h as recommended by the manufacturer of Edu detection kits (Ribobio, Guangzhou, China).

**RNA extraction, library construction and transcriptome sequencing**

Total RNA was extracted from testis tissues using Trizol (Invitrogen, Carlsbad, CA, USA) according to the manual instructions. The total RNA was qualified and quantified with a Nano Drop and Agilent 2100 bioanalyzer (Thermo Fisher Scientific, MA, USA). The ribosomal RNA (rRNA) was first removed using target-specific oligos and RNase H reagents to deplete both cytoplasmic (5S rRNA, 5.8S rRNA, 18S rRNA and 28S rRNA) and mitochodrial ribosomal RNA (12S rRNA and 16S rRNA) from the RNA preparations. The RNA was fragmented into small pieces using divalent cations under elevated temperature after SPRI beads purification. The cleaved RNA fragments were copied into first strand cDNA with reverse transcriptase and random primers, and followed by second strand cDNA synthesis with DNA Polymerase I and RNase H. This process was to remove the RNA template and synthesizes a replacement strand, incorporating dUTP in place of dTTP to generate ds cDNA. The cDNA fragments then had the addition of a single 'A' base and subsequent ligation of the adapter. Following UDG treatment, the incorporation of dUTP quenched the second strand during amplification. The products were enriched with PCR to create the cDNA library. The libraries were assessed quality and quantity by checking the distribution of the fragments size using the Agilent 2100 bioanalyzer and quantifying the library using real-time quantitative PCR (TaqMan Probe). The qualified libraries were sequenced pair end on the BGISEQ-500/ MGISEQ-2000 System (BGI-Shenzhen, China). Then sequencing reads filtering genome mapping, gene expression analysis and differentially expressed genes (DEG) detection were performed.

**Heatmap analysis**

According to the results of differential gene detection (Fold Change≥1.50), the R package pheatmap (version 1.0.12) of R 4.0 was used to perform the heat maps. Due to the large variation in these DEGs, the expression values of the samples were treated with log2 in the data analyses of RNAseq.

**Western blotting**

Western blotting (WB) analysis was performed as previously described (Han et al. 2015). The following primary antibodies were used: Sox30 rabbit polyclonal antibody (1:1000, ab26024, Abcam), Rec8 rabbit monoclonal antibody (1:1200, ab192241, Abcam), Stra8 rabbit polyclonal antibody (1:1000, ab49602, Abcam), γ-H2A.X rabbit monoclonal antibody (1:1000, #9718, Cell Signaling Technology, Boston, MA, USA), Cyp26b1 rabbit polyclonal antibody (1:1000; 21555-1-AP, proteintech, Inc, IL,USA ), ALDH1 goat polyclonal antibody (1:1000, sc-22591, Santa Cruz Biotechnology), ALDH2 mouse monoclonal antibody (1:1000, sc-68348, Santa Cruz Biotechnology), Foxl2 rabbit polyclonal antibody (1:800, sc-22591, Santa Cruz Biotechnology), Wnt4 mouse monoclonal antibody (1:1000, sc-376279, Santa Cruz Biotechnology), Ctnnb1 mouse monoclonal antibody (1:1000, sc-7963, Santa Cruz Biotechnology), Rspo1 rabbit polyclonal antibody (1:800; ab106556, Abcam) and Sox9 rabbit polyclonal antibody (1:800; GB11280, servicebio, Wuhan, China). Secondary (anti-rabbit, anti-mouse and anti-goat) antibodies were horseradish peroxidase (HRP)-conjugated.

**Immunofluorescence**

Testis tissues were harvested into ice-cold PBS, fixed with freshly prepared 4% paraformaldehyde for 30 min and perforated with a needle. After overnight fixation, the tissues were incubated in 15% sucrose for 1 day and in 30% sucrose for another 1 day. The tissues were frozen in liquid cooled isopentane. The sections (5µm) were obtained and placed onto slides (Fisherbrand Colorfrost Plus), air-dried. The section slides were washed with PBS and permeabilized with 0.1% Triton X-100 for 1h. These section slides were then quenched with 0.1 M glycine, washed with PBS, blocked with 10% fetal bovine serum, incubated with primary antibodies and fluorescent secondary antibodies. The primary antibodies: Sox30 rabbit polyclonal antibody (1:100, ab26024, Abcam), Sox30 rabbit polyclonal antibody(1:60, Santa Cruz Biotechnology, sc-20104), Rec8 rabbit monoclonal antibody (1:100, ab192241, Abcam), Stra8 rabbit polyclonal antibody (1:100, ab49602, Abcam), γ-H2A.X rabbit monoclonal antibody (1:100, #9718, Cell Signaling Technology, Boston, MA, USA), Sycp3 (Scp3) mouse monoclonal antibody (1:100, sc-74569, Santa Cruz Biotechnology), Cyp26b1 rabbit polyclonal antibody (1:80, 21555-1-AP, proteintech), ALDH1 goat polyclonal antibody (1:100, sc-22591, Santa Cruz Biotechnology) and ALDH2 mouse monoclonal antibody (1:100, sc-100496, Santa Cruz Biotechnology), Foxl2 rabbit polyclonal antibody (1:80, sc-22591, Santa Cruz Biotechnology), Ctnnb1 mouse monoclonal antibody (1:100, sc-7963, Santa Cruz Biotechnology), Rspo1 rabbit polyclonal antibody (1:80; ab106556, Abcam). The fluorescent secondary antibodies: DyLight 549 Goat Anti-Rabbit IgG (1:100, A23320, Abbkine, Inc, CA, USA), DyLight 488 Goat Anti-Mouse IgG (1:100, A23210, Abbkine), DyLight 405 Rabbit Anti-Goat IgG (1:100, A23130, Abbkine).

**Retinoic acid concentration**

Retinoic acid (RA) concentration in testis was determined using mouse retinoic acid ELISA Kit (MyBioSource, San Diego, CA, USA MBS706971_48T) according to the directions. Briefly, testes were dissected, weighed and cut into pieces in ice PBS (100mg testis tissue added in 500µm PBS with protease inhibitor). The reagents and samples were prepared as instructed. A blank without any solution was set. Total 50µl standard or sample was added to each well and then 50µl HRP-conjugate (1x) was added to each well (not to blank well), which was incubated 40 minutes at 37 °C. These samples were aspirated and washed for 5 times. 90µl TMB substrate was added to each well, and incubated for 20 minutes protect from light at 37 °C. Finally, 50µl stop solution was added to each well and read at 450nm.

**Construction of vectors and cell transfection**

The vector of full-length Sox30 open reading frame was constructed by synthesis and PCR amplification. Briefly, single stranded oligonucleotides were designed and synthesized. The synthetic oligonucleotides were spliced into complete sequence by PCR, validated by sequencing, and then subcloned into the pIRES2-EGFP expression vector (Invitrogen Preservation, Carlsbad, CA, USA) and validated by sequencing. The full-length *Cyp26b1*, *Rec8*, *Stra8*, *Sox9*, *Wnt4*, *Rspo1*, *Foxl2* and *Ctnnb1* promoters were constructed into pGL3-basic vector by PCR amplification. Briefly, the full-length promoters of these genes were amplified from genomic DNA of normal NIH3T3 cells by PCR and validated by direct sequencing. They were then sub-cloned into the pGL3-basic vector and further confirmed by sequencing.

**Site-directed mutagenesis assay**

Sox30 HMG-box constructs were mutated using a QuikChange Lightning Site-Directed Mutagenesis Kit (Stratagene, La Jolla, CA, USA) as described (Han et al. 2018b). Briefly, mutagenic primers were designed, and the mutant strand was synthesized by RT-PCR. The amplification product was digested by Dpn I, and then was transformed into XL10-Gold Ultracompetent cells. The mutations were validated by direct sequencing.

**Luciferase reporter assay**

The NIH3T3 and/or HEK293 cells were plated in 24-well plates (3.5×10^4^ cells per well) in triplicate for each condition. After overnight incubation, the cells were transfected with a DNA mixture containing pGL3-Cyp26b1/Rec8/Stra8/Sox9/Wnt4/Rspo1/ Foxl2/Ctnnb1 promoter-luciferase, wild-type pIRES2-EGFP-Sox30/mutated pIRES2- EGFP-Sox30/empty vector, and pRL-TK plasmids by ViaFect Transfection Reagent (Promega). The NIH3T3 and HEK293 cells were lysed with passive lysis buffer at 36h post-transfection, and luciferase activities were measured by the fluorescence microplate reader measurement system Varioskan LUX (Thermo Fisher, Waltham, MA, USA) using a Dual-luciferase reporter kit (Promega) according to the direction. Each experiment was performed in triplicate and repeated three times.

**Chromatin immunoprecipitation-PCR assay**

The ChIP analyses were performed using a tissue ChIP Assay Kit (Epigentek, Bi County Blvd. Ste., Farmingdale, NY, USA, P-2003) or a cell ChIP Assay Kit (Pierce, Rockford, lL, USA, 26156) according to the directions. Briefly, for the tissue ChIP Assay Kit testis tissues were disaggregated and cross-linked in a final concentration of 1% formaldehyde. The disaggregated tissue pellets were lysed, and the DNA was sheared. The sheared DNA was immunoprecipitated with Sox30 antibody. Then the DNA of IP was eluted and purified. For the cell ChIP Assay Kit, 2x10^6^ NIH3T3 cells were cross-linked in a final concentration of 1% formaldehyde. The cross-linked cells were lysed and digested with micrococcal nuclease. The digested DNA was immunoprecipitated with Sox30 antibody after analysis of chromatin digestion. Then, the DNA of IP was eluted and purified. The immunoprecipitated and input DNA samples were used as templates for RT-PCR analysis using the primers listed in Supplemental Table S2.

**Statistical analysis**

Statistical analyses were performed using SPSS 15.0 software (SPSS, Inc., Chicago, IL, USA). The data are expressed as mean ± standard error of mean (SEM). The differences between two or three groups were analyzed using Student’s t-test, Tukey test and One-way ANOVA (the data meet the assumptions of the tests). The p-values of less than 0.05 were considered statistically significant. The exact times, condition and sample size of each experiment are described in the figure legends. The animals were grouped randomly, and data were independently analyzed at least by two pathologists or statistical experts.
